# Dysregulated microRNA–mRNA networks in SOD1^G93A^ skeletal muscle reveal metabolic impairments in amyotrophic lateral sclerosis

**DOI:** 10.1242/dmm.052963

**Published:** 2026-10-09

**Authors:** Libby Moody, Eleni Christoforidou, Greig Joilin, Andrew Dilley, Majid Hafezparast

**Affiliations:** ^1^ Department of Neuroscience, School of Life Sciences, University of Sussex, Brighton BN1 9QG, UK; ^2^ Department of Clinical Neuroscience, Brighton and Sussex Medical School, University of Sussex, Brighton BN1 9PX, UK

**Keywords:** Amyotrophic lateral sclerosis, Motor neurone disease, SOD1, Muscle, MicroRNA, Metabolism

## Abstract

Amyotrophic lateral sclerosis (ALS) is a multi-system disease in which skeletal muscle actively contributes to pathology, yet the regulatory circuits that drive muscle dysfunction remain unclear. We examined miRNA–mRNA interactions in the gastrocnemius of hSOD1^G93A^ mice across pre-, early- and late-symptomatic stages, using RNA sequencing, bioinformatics and reverse transcription quantitative PCR (RT-qPCR). Compared with hSOD1^WT^ and non-transgenic control muscle, hSOD1^G93A^ muscle showed mutation-specific transcriptome reprogramming: 48 dysregulated miRNAs and 558 mRNAs at pre-symptomatic, and 64 miRNAs and 685 mRNAs at late-symptomatic, stages. Functional enrichment pinpointed carbohydrate-handling pathways (glycolysis/gluconeogenesis, pentose-phosphate, fructose–mannose metabolism) as the dominant downregulated gene sets. Network analysis revealed clusters in which upregulated miRNAs converged on, and showed inverse expression patterns relative to metabolic transcripts. RT-qPCR confirmed inverse expression of ten candidate miRNAs and 11 metabolic mRNAs, substantiating miRNA-guided repression of glycolytic enzymes and energy-sensing nodes. Collectively, we show that SOD1^G93A^ drives an early, sustained miRNA signature that dampens glycolysis gene expression, which could promote the fast-to-slow fibre-type transition and exacerbate energy deficit in ALS muscle. Targeting these circuits offers a strategy to restore metabolic balance and slow disease progression.

## Teaser

**Summary:** This study identifies microRNA–mRNA networks that disrupt muscle energy metabolism in a mouse model of amyotrophic lateral sclerosis, revealing early glycolytic suppression and highlighting therapeutic targets to restore metabolic balance.

## INTRODUCTION

Amyotrophic lateral sclerosis (ALS) is a fatal, progressive neurodegenerative disorder characterised by the selective loss of upper and lower motor neurons (MNs). This neurodegeneration leads to muscle weakness, atrophy and, ultimately, paralysis, with patients typically succumbing to respiratory failure within 2–5 years of symptom onset (Kiernan et al., 2011). Despite extensive research, the underlying molecular mechanisms of ALS remain poorly understood, and there are no effective therapies that can halt or reverse disease progression. Consequently, there is an urgent need to uncover the cellular processes and molecular drivers that contribute to ALS pathogenesis.

Genetic factors play a critical role in both familial and sporadic forms of ALS, with over 150 ALS-associated variants identified in the Cu/Zn superoxide dismutase 1 (*SOD1*) gene in 12–23.5% of familial cases and up to 7.3% of sporadic cases (Rosen et al., 1993; Andersen, 2006). Most of these variants are missense mutations and some are nonsense mutations that alter the length of the final polypeptide, but none are believed to prevent production of the protein, suggesting that aberrant gain-of-function characteristics acquired by the mutant protein render it prone to aggregation and, ultimately, the development of ALS (Ghasemi and Brown, 2018). The hSOD1^G93A^ transgenic mouse model, in which a glycine-to-alanine substitution at codon 93 of the human *SOD1* gene is expressed, has been extensively used to mimic many aspects of ALS pathology, including MN degeneration, neuromuscular junction (NMJ) disruption and muscle atrophy.

Beyond the degeneration of MNs, emerging evidence suggests that skeletal muscle is not merely a passive victim of denervation but may also contribute actively to disease pathology (Shefner et al., 2023). Direct evidence that skeletal muscle can initiate MN pathology has been provided by transgenic mice in which expression of the ALS-associated G37R or G93A human SOD1 variants was restricted to skeletal muscle. Despite the absence of human SOD1 expression in the central nervous system, these mice developed severe myopathy and NMJ abnormalities, followed by distal motor axonopathy, degeneration and loss of lumbar spinal MNs, and progressive motor impairment (Wong and Martin, 2010). Subsequent longitudinal characterisation of these mice demonstrated progressive weakness, paralysis, shortened lifespan and a fatal ALS-like phenotype, supporting a non-cell-autonomous mechanism in which muscle pathology promotes retrograde MN degeneration (Martin and Wong, 2020). Furthermore, independent studies using the muscle-specific MLC/SOD1^G93A^ model further demonstrated that mutant SOD1 expression within muscle is sufficient to cause oxidative stress, mitochondrial dysfunction and severe myopathy, followed by structural abnormalities of presynaptic motor terminals and NMJ dismantlement (Dobrowolny et al., 2008, 2018b). Complementary compartmentalised culture studies showed that SOD1^G93A^-expressing myocytes release factors that disrupt motor axons and NMJs and impair mitochondrial axonal transport, ultimately triggering MN death (Martínez et al., 2024; Maimon et al., 2018). Together, these findings support a non-cell-autonomous mechanism through which mutant-SOD1-associated muscle pathology can contribute to distal axonal and MN degeneration.

In ALS, skeletal muscle undergoes significant metabolic reprogramming that is characterised by alterations in energy homeostasis, mitochondrial dysfunction, oxidative stress, and a transition from a fast-twitch to a slow-twitch fibre phenotype, which precede detectable MN loss or overt denervation (Dupuis et al., 2003; Palamiuc et al., 2015). For example, in the hSOD1^G93A^ model of ALS, muscle remodelling and mitochondrial defects manifest before clinical onset (Scaricamazza et al., 2020). In hSOD1^G93A^ transgenic animals, muscle-specific metabolic shifts such as increased lipid utilisation and inhibition of pyruvate oxidation occur independently of MN degeneration, arguing that muscle-intrinsic metabolic derangement is upstream of denervation (Scaricamazza et al., 2020). Moreover, people with ALS and animal models show systemic hypermetabolism, weight loss, altered glucose/lipid handling and shifts in substrate use, further implicating energy-homeostasis failure as an early event (Anakor et al., 2022).

These early muscle alterations may instigate a pathogenic retrograde signalling cascade that impairs NMJ maintenance and exerts stress on motor axons and cell bodies. Indeed, muscle can secrete a variety of trophic, metabolic or stress-related signals (e.g. myokines, extracellular vesicles) that influence MN health and NMJ stability (Anakor et al., 2022; Le Gall et al., 2022). Dysfunctional muscle, via altered agrin/MuSK signalling, aberrant synaptic gene expression or intrinsic secretome changes, has been shown to compromise NMJ integrity even in the absence of MN pathology, suggesting that muscle defects can drive synaptic destabilisation and subsequent ‘dying-back’ axonopathy (Lépine et al., 2022; Anakor et al., 2022). For instance, skeletal muscle from people with ALS or myocytes carrying ALS variants exhibit intrinsic deficits in acetylcholine receptor clustering, agrin/MuSK response or NMJ stability (Lépine et al., 2022). In mouse models, muscle-restricted perturbations (e.g. BET1L knockdown) exacerbate NMJ degeneration and MN loss, underscoring the potential of muscle-originated signals to drive MN pathology (Eckardt et al., 2025). Thus, metabolic stress, reactive oxygen species or secreted mediators from muscle may impair the local synaptic milieu, compromise axonal transport or trigger local degeneration cascades in motor axons.

In this context, microRNAs (miRNAs) emerge as plausible mediators linking muscle metabolic stress to NMJ dysfunction and MN injury. miRNAs are post-transcriptional regulators of gene expression that can act as important modulators of muscle metabolism and regeneration (Dobrowolny et al., 2018a). In muscle, miRNAs such as miR-206 have been implicated in promoting NMJ repair and muscle reinnervation following injury, and their dysregulation could thus weaken the synaptic support environment in ALS (Toivonen et al., 2014; Williams et al., 2009). More recently, other muscle-derived miRNAs (e.g. miR-126) have been shown to be loaded into extracellular vesicles, transported to the presynaptic compartment and modulate local protein synthesis at NMJs (e.g. suppression of TDP-43 accumulation in axons and delay of NMJ disruption) (Ionescu et al., 2025). Dysregulation of such miRNAs in ALS could, therefore, impair the muscle-to-neuron crosstalk necessary to maintain NMJ integrity, exacerbate synaptic destabilisation and, ultimately, contribute to MN degeneration.

Therefore, the present study investigates the hypothesis that ALS-associated variants lead to aberrant miRNA expression in skeletal muscle, which, in turn, disrupts downstream mRNA networks that regulate muscle function. By profiling the miRNA and mRNA transcriptomes in the gastrocnemius muscle of male transgenic mice carrying the human *SOD1* gene with the ALS-linked *G93A* variant (referred to as hSOD1^G93A^), and comparing these with miRNA and mRNA transcriptomes in the gastrocnemius muscle of male transgenic mice carrying the wild-type human *SOD1* gene (referred to as hSOD1^WT^) and non-transgenic (NTg) controls, we aimed to distinguish changes driven by the pathogenic variant from those caused by overexpression of the *SOD1* transgene. The gastrocnemius was selected because it is a large, well-characterised hindlimb muscle that provided sufficient tissue for parallel miRNA and mRNA profiling and subsequent reverse transcription quantitative PCR (RT-qPCR) validation from the same muscle. It has a heterogeneous but predominantly fast-twitch fibre composition, comprising mainly type II fast glycolytic and fast oxidative fibres, with a smaller population of type I slow oxidative fibres. Published estimates in mouse gastrocnemius indicate ∼55% fast-fatigable, 32% fast fatigue-resistant and 8% slow fibres, although fibre composition varies spatially across the muscle, with the superficial regions being particularly enriched in type IIb glycolytic fibres (Atkin et al., 2005; Bloemberg and Quadrilatero, 2012). This predominantly fast composition is relevant to the hSOD1^G93A^ model because fast motor units innervating the tibialis anterior, extensor digitorum longus and medial gastrocnemius are lost earlier than those innervating the more slowly contracting soleus (Hegedus et al., 2007).

Using next-generation sequencing combined with bioinformatic analyses, we identified significant dysregulation of miRNAs and mRNAs in the ALS muscle that we further validated by RT-qPCR in a larger sample size. Functional enrichment and network analyses pointed to disruption of key pathways involved in carbohydrate metabolism and energy homeostasis. These findings provide insight into the molecular mechanisms underlying metabolic impairment in ALS skeletal muscle and highlight potential targets for therapeutic intervention aimed at mitigating muscle atrophy and improving outcomes for people with ALS.

## RESULTS

### RNA and sequencing integrity

To obtain an overview of miRNA–mRNA expression dysregulation in ALS muscle, we profiled the miRNA and mRNA transcriptomes of gastrocnemius muscle tissue from hSOD1^WT^, hSOD1^G93A^ and NTg mice throughout disease progression. As expected, the mass of the hSOD1^G93A^ muscles was significantly lower than that of the control groups, across all timepoints, owing to disease-associated muscle atrophy (Fig. 1A). Interestingly, the mass of the muscle tissues from the hSOD1^WT^ control group was also significantly lower than that of the muscle tissues from the NTg group across all timepoints (*P*=0.0108), suggesting that the overexpression of hSOD1^WT^ causes mild muscular atrophy. However, comparison between genotypes at the specific timepoints revealed that there was only a significant difference between the control groups at the 120-day timepoint (*P*=0.0033).

**Fig. 1.**
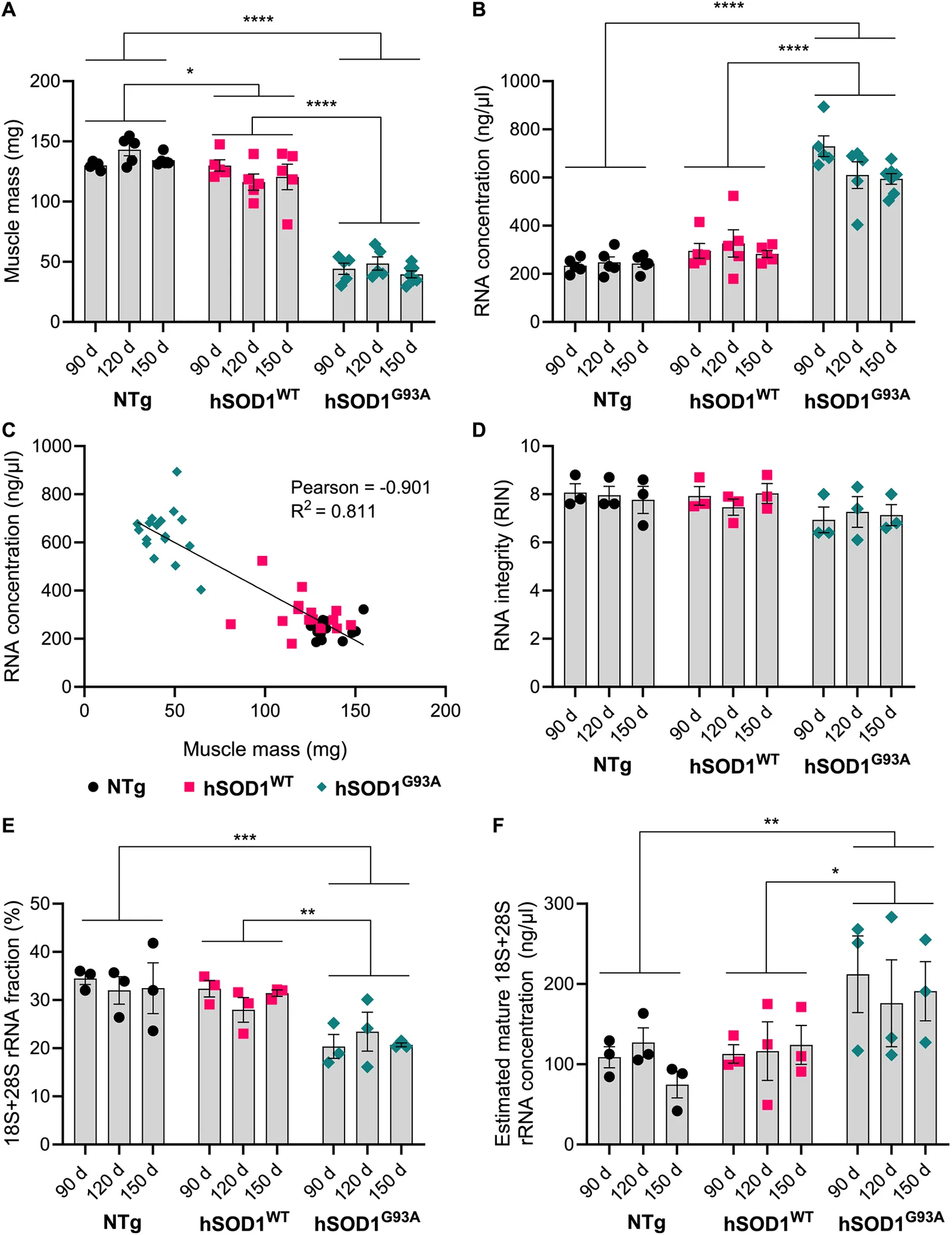
**Extraction of RNA from mouse gastrocnemius muscle.** The whole gastrocnemius muscle was collected from male transgenic mice carrying the wild-type human *SOD1* gene (referred to as hSOD1^WT^) or the human *SOD1* gene with the ALS-linked *G93A* variant (referred to hSOD1^G93A^) and non-transgenic (NTg) mice at 90, 120 and 150 days old and lysed, then RNA was extracted from a volume of tissue lysate equivalent to a 30 mg input mass. (A) Muscle mass prior to RNA extraction. (B) Concentration of total RNA obtained from each sample after RNA extraction. (C) Correlation between muscle mass prior to RNA extraction and final extracted RNA concentration. (D) RNA integrity. (E) Mature 18S+28S rRNA peak fraction, calculated as the sum of the percentages of total Bioanalyzer electropherogram area occupied by the 18S and 28S peaks. (F) Estimated mature 18S+28S rRNA concentration in the original total RNA eluate, calculated by multiplying the total RNA concentration by the combined 18S+28S peak fraction. Excluding C, data are shown as mean±s.e.m. and were analysed by two-way ANOVA with Tukey's post-hoc. *N*=5–7 biological replicates per genotype per age. d, days; RIN, RNA integrity number. *P<0.05, **P<0.01, ***P<0.001, ****P<0.0001.

Using an unbiased approach to obtain RNA representative of the entire tissue, it was expected that the final concentration of RNA obtained from each sample would be similar across all genotypes. However, quantification of RNA concentration revealed that the hSOD1^G93A^ samples contained a significantly higher concentration of RNA than that of both control groups (Fig. 1B), with a strong negative correlation identified between the original muscle mass and the RNA concentration (Pearson's ρ=‒0.9, *P*<0.0001, Fig. 1C). This indicated that hSOD1^G93A^ gastrocnemius tissue contained significantly more total RNA per mg of tissue, which is an interesting observation that suggests a global increase in RNA expression in ALS muscle. RNA integrity number (RIN) values ranged from 6.1 to 8.8, and there were no statistically significant differences in RNA integrity between genotypes (Fig. 1D), showing that the higher concentration of RNA obtained from the hSOD1^G93A^ muscles was not an artefact of degraded RNA.

Interestingly, the greater total RNA concentration from hSOD1^G93A^ muscle was accompanied by a significant increase in the estimated concentration of mature 18S and 28S ribosomal RNA (rRNA) calculated from the electropherogram peaks produced by the Bioanalyzer (hSOD1^G93A^ versus hSOD1^WT^, *P*=0.0274; hSOD1^G93A^ versus NTg, *P*=0.0087; Fig. 1E). This suggests that increased mature rRNA contributes, at least partly, to the greater total RNA concentration observed in the mutant muscle. However, the combined 18S+28S peak areas represented a significantly smaller fraction of the total RNA electropherogram area in hSOD1^G93A^ muscle than in either control genotype (hSOD1^G93A^ versus hSOD1^WT^, *P*=0.0024; hSOD1^G93A^ versus NTg, *P*=0.0002; Fig. 1F). Thus, mature rRNA was increased in absolute concentration but was not selectively or disproportionately enriched, indicating that non-18S/28S RNA species made an even greater proportional contribution to the expanded RNA pool. These findings are compatible with altered RNA and ribosome homeostasis in hSOD1^G93A^ muscle but do not establish defective ribosome biogenesis or abnormal translation. The Bioanalyzer analysis measures mature 18S and 28S rRNA abundance indirectly and cannot assess precursor-rRNA (pre-rRNA) transcription or processing, ribosome assembly, polysome abundance or protein-synthesis rates. Direct investigation of these processes would require measurements such as pre-rRNA analysis, ribosome profiling, polysome analysis or protein-synthesis assays, which would be interesting to investigate in future studies.

### Differential miRNA and mRNA expression within hSOD1^G93A^ muscle

Muscle RNA was used to prepare miRNA and mRNA libraries for next-generation sequencing, from three mice per genotype and timepoint. The quality control of the miRNA and mRNA sequencing reads, and the mapping information are shown in Figs S1 and S2, respectively. To explore differential miRNA and mRNA expression following next-generation sequencing, the DESeq2 package in R was used to normalise the reads to sequencing depth and subsequently compare expression between genotypes and across timepoints. The miRNA profiles of NTg and hSOD1^WT^ samples were similar at all timepoints, and no miRNAs were found to be significantly dysregulated between these groups. The same was true for the mRNA expression levels. This suggests that overexpression of the hSOD1^WT^ transgene does not influence expression of miRNAs or mRNAs in the gastrocnemius muscle, at the timepoints investigated in this study.

In contrast, the hSOD1^G93A^ variant caused significant dysregulation of muscle miRNAs and mRNAs when compared to the NTg and hSOD1^WT^ samples. Notably, 48 miRNAs were significantly dysregulated in the hSOD1^G93A^ samples compared to those in both control groups at 90 days and 120 days, and this increased to 64 miRNAs at 150 days (Fig. 2A–C). Similar numbers of miRNAs were upregulated as were downregulated. At each timepoint, only a handful of miRNAs were found to have significantly elevated or reduced expression compared to that in one, but not both, of the two control groups, which likely reflects the similarities in the miRNA expression profiles of the two control groups. Similarly, compared to those in both control groups, 558 mRNAs were dysregulated at 90 days, 544 mRNAs were dysregulated at 120 days, and 685 mRNAs were dysregulated at 150 days (Fig. 2D–F). Notably, 433 of these mRNAs were significantly dysregulated at all three timepoints, compared to those in both controls (180 upregulated, 253 downregulated).

**Fig. 2.**
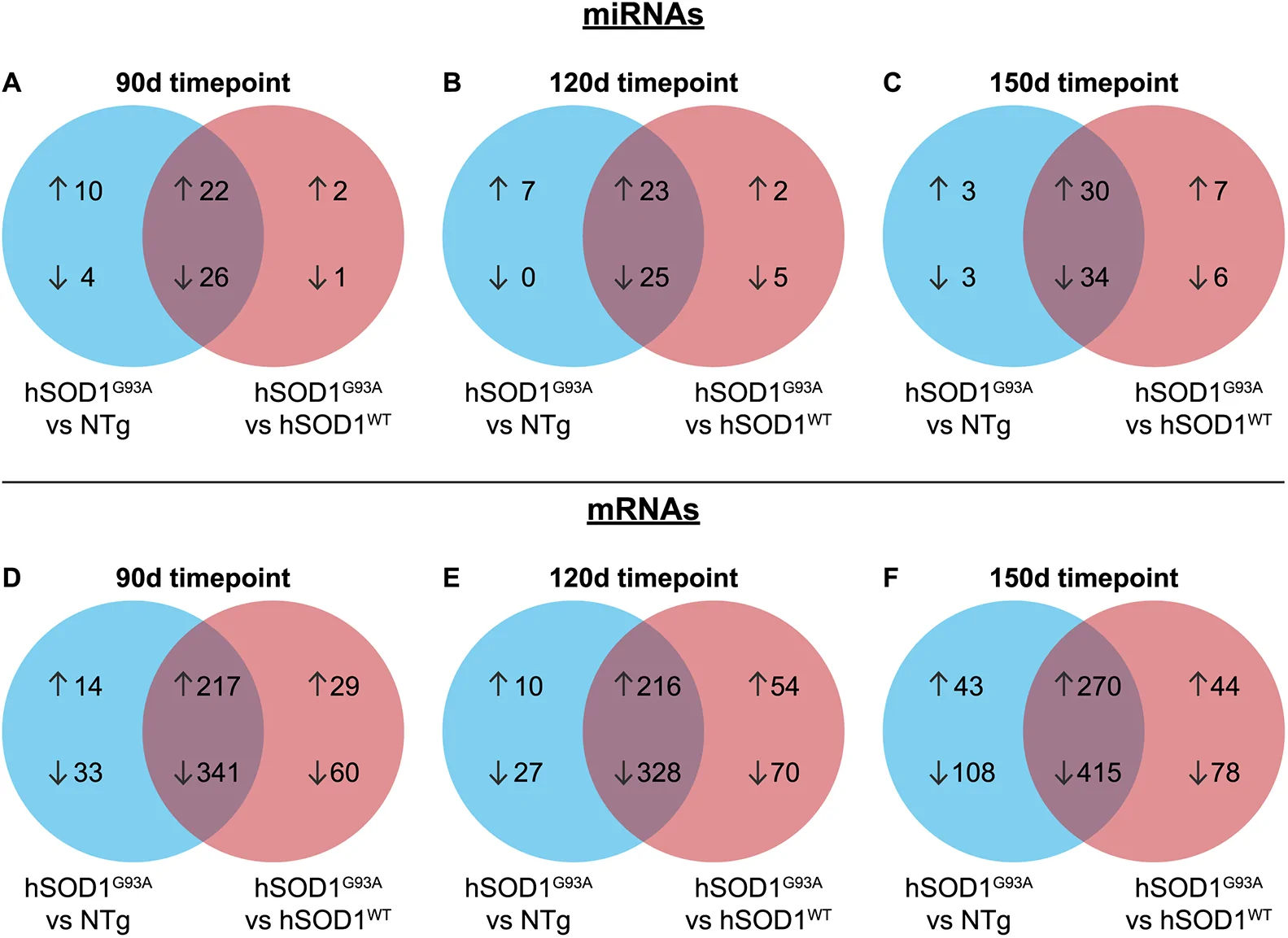
**Differential expression of miRNAs and mRNAs in hSOD1^G93A^ compared to hSOD1^WT^ and NTg gastrocnemius muscle.** Quantification of miRNA and mRNA levels in muscle tissue was performed using next-generation sequencing. (A–F) Number of significantly dysregulated miRNAs (A–C) and mRNAs (D–F) between the hSOD1^G93A^ and NTg groups, and between the hSOD1^G93A^ and hSOD1^WT^ groups, separated by timepoint. Data from samples collected at the 90-day timepoint (A,D), the 120-day timepoint (B,E) and the 150-day timepoint (C,F). ‘Up’ arrows represent the number of upregulated miRNAs, whereas ‘down’ arrows indicate the number of downregulated miRNAs. *N*=3 mice per genotype per timepoint.

Overall, fold changes for miRNAs ranged from +17.5 to −4.1, with miR-206-3p and miR-34c-5p consistently the most upregulated miRNAs at all three timepoints, and miR-29c-5p and miR-181a-2-3p consistently the most downregulated. Fold changes for mRNAs were larger, ranging from +248 to −116. In contrast to miRNAs, at each timepoint, and across all timepoints, more mRNAs were downregulated than upregulated. The full list of significant miRNA expression changes between the hSOD1^G93A^ samples and each control group, at each timepoint, is shown in Tables S1 and S2, and for mRNAs in Tables S3 and S4.

To explore changes in muscle miRNAs over time, their expression profiles were compared between the 90-day and 150-day timepoints, within each genotype. Minimal miRNA dysregulation was identified between these timepoints. There were no miRNAs for which expression was significantly changed over time in the hSOD1^G93A^ genotype group and not also changed in one or both other genotype groups, suggesting that there were no changes over time caused by the ALS-associated variant. Across all three genotypes, 15 miRNAs were significantly downregulated at 150 days compared to 90 days, and one miRNA was upregulated at 150 days, likely reflecting age-associated changes in the expression of these miRNAs.

Subsequently, the same analysis on the mRNA data revealed that there were no significant changes to mRNA expression in the hSOD1^WT^ group over time, and only one mRNA was upregulated in the NTg group over time – radical S-adenosyl methionine domain containing 2 (*Rsad2*). In healthy mice with a life expectancy of ∼24 months, the timeframe from 90 to 150 days is small, which likely explains the minimal change to mRNA expression in the control groups. However, minimal mRNA expression change over time was also found in the hSOD1^G93A^ muscle, in which these timepoints reflect a significant proportion of the lifespan, given that hSOD1^G93A^ mice die shortly after 150 days. Between 90 days and 150 days, only 11 mRNAs were downregulated, and one mRNA was upregulated. This was unexpected owing to the severe atrophy of the muscle seen throughout disease progression and perhaps suggests that the differences in gene expression in the hSOD1^G93A^ mouse are ‘built-in’ from before the earliest timepoint investigated in this study. The full list of significant miRNA expression changes within each genotype, between the 90-day and 150-day timepoints, is shown in Table S5, and for mRNAs in Table S6.

### Functional enrichment analysis of miRNAs and mRNAs dysregulated in the hSOD1^G93A^ muscle

We used three web-based tools to explore possible target genes of the muscle miRNAs that were found to be dysregulated (agnostic to direction) in the hSOD1^G93A^ muscle samples, compared to those in samples from both controls, at all timepoints. First, miRPath identified 108 enriched Kyoto Encyclopedia of Genes and Genomes (KEGG) pathways using both TarBase ‘direct confirmed’ mRNA targets and miRTarBase ‘strongly evidenced’ validated targets. MiRWalk identified 22 enriched KEGG pathways, 17 of which were also in common with miRPath. As these 17 enriched pathways were identified using a combination of experimentally validated targets and algorithm predictions, they were considered to be very robust. In contrast, miRSystem identified 15 enriched KEGG pathways that were different from those identified with miRPath and miRWalk. Interrogating three databases, each with different stringency and enrichment identification methods, provided a broader, more comprehensive overview of potential pathway enrichments associated with the dysregulated miRNAs, summarised in Fig. 3A.

**Fig. 3.**
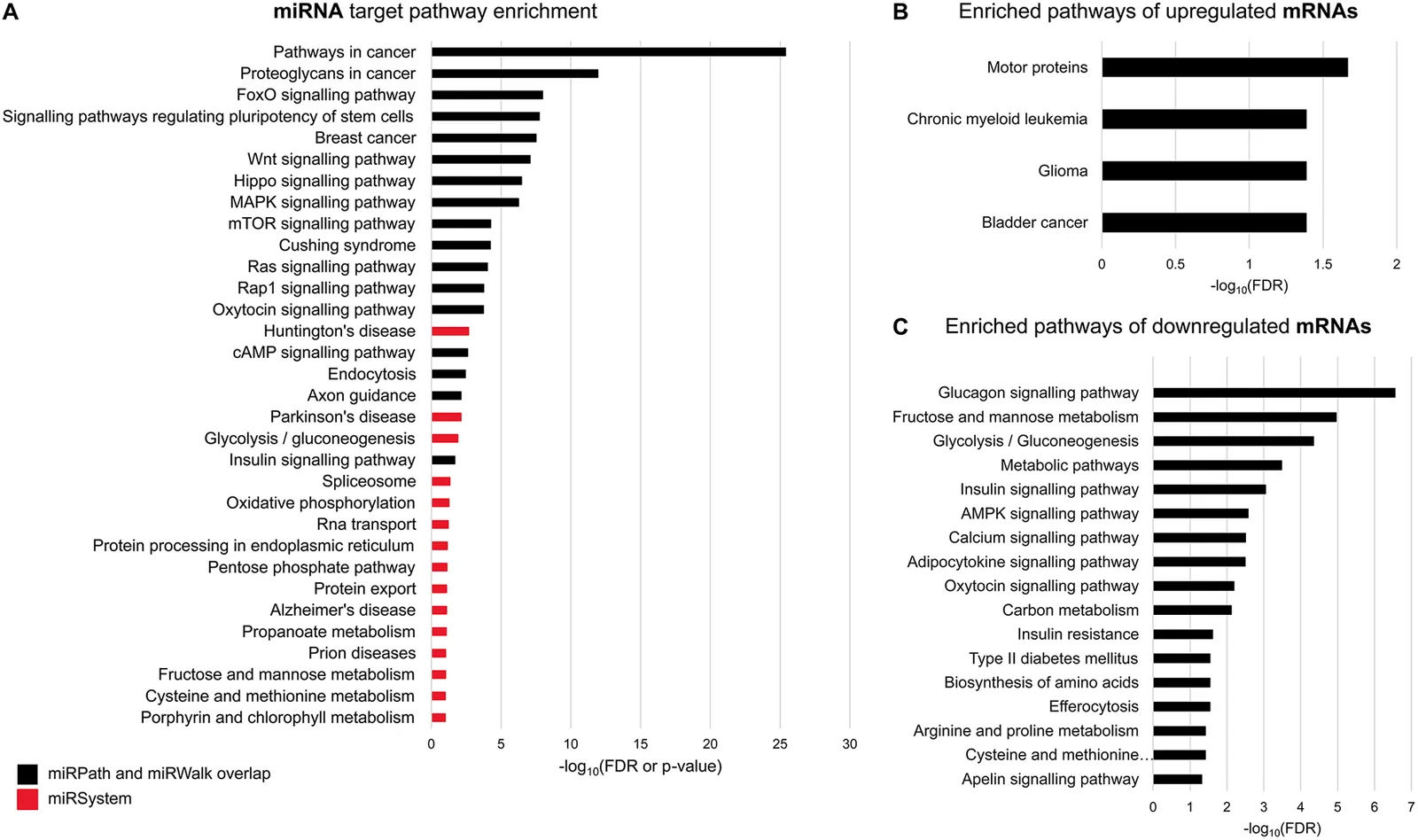
**Enriched pathways targeted by dysregulated miRNAs and of dysregulated mRNAs in the hSOD1^G93A^ muscle.** (A) All miRNAs found to be significantly dysregulated at all three timepoints in hSOD1^G93A^ muscles, compared to muscles in both control groups, were run through three different enrichment tools to identify biological pathway enrichments of the predicted and validated miRNA targets. Enriched Kyoto Encyclopedia of Genes and Genomes (KEGG) pathways from both miRPath and miRWalk are shown in black; enriched KEGG pathways from miRSystem are shown in red. The −log10(FDR) value from miRPath and the –log10(*P*-value) from miRSystem are plotted. (B,C) Enriched pathways of mRNAs found to be significantly upregulated (B) or downregulated (C), at all three timepoints in hSOD1^G93A^ muscles, compared to muscles in both control groups (DAVID enrichment analysis). The −log10(FDR) values are plotted. FDR, false discovery rate.

Next, the DAVID gene enrichment tool was used to interrogate the mRNAs that were consistently upregulated or downregulated in the hSOD1^G93A^ muscle at all three timepoints, compared to those in both controls, to gain insight into directional enrichments. Only four KEGG pathways were significantly enriched from the input list of upregulated mRNAs, including the motor protein pathway (Fig. 3B). In contrast, 17 KEGG pathways were enriched amongst the downregulated mRNAs, including many pathways related to carbohydrate metabolism, amino acid metabolism and the calcium signalling pathway (Fig. 3C). This suggests a major energy deficit and downregulation of anabolic processes in the hSOD1^G93A^ muscle, which does not appear to be compensated for by upregulation of alternative metabolic pathways. The AMPK pathway was also enriched among downregulated mRNAs, which included the mRNAs encoding GLUT4 (also known as SLC2A4) and AMPK, which directly influence glycolysis. Further corroborating this, four of the top five functional annotation clusters generated by DAVID using the list of downregulated mRNAs as input included terms related to carbohydrate metabolism.

To assess changes during disease progression, we repeated the functional enrichment analysis for both the miRNAs and mRNAs separately for each of the three timepoints. The mRNA analysis showed that suppression of metabolic pathways was already present at 90 days and persisted across all timepoints, with broader metabolic, stress response and immune involvement at 150 days (Tables S7–S12). For miRNAs, we likewise identified substantial overlap between ages, including recurrent enrichment of AMPK, PI3K–Akt, MAPK, FoxO, mTOR autophagy and apoptosis pathways (Tables S13–S19). Together, these findings suggest that the major molecular abnormalities in hSOD1^G93A^ muscle are established early and remain relatively stable during disease progression, with late-stage disease characterised mainly by expansion of the existing metabolic and stress response signature rather than the emergence of an entirely distinct regulatory programme.

### Dysregulated miRNA–mRNA networks

Some KEGG pathways were functionally enriched in both the miRNA and mRNA functional enrichment analyses. Glycolysis/gluconeogenesis, fructose and mannose metabolism, cysteine and methionine metabolism and oxytocin signalling pathways were enriched in both the dysregulated miRNA input and the downregulated mRNA input, both from across all three timepoints. This overlap suggests that the miRNAs identified as aberrantly expressed in the hSOD1^G93A^ muscle directly downregulate the genes in these pathways. Thus, to further explore significant interactions between the dysregulated miRNAs and mRNAs, Cytoscape was used with the CyTargetLinker plugin. This platform allows visualisation of interactions between selected miRNAs and mRNAs, using functional annotation data from the TarBase database of confirmed direct interactions and the TargetScan database of predicted interactions.

First, a network was created to visualise any interactions between the miRNAs that were upregulated in the hSOD1^G93A^ muscle at all timepoints, against the mRNAs that were downregulated at all timepoints. As the input was rather large, the interactions were filtered to show only those annotations that were present in both the TargetScan and miRTarBase databases. The resulting network included eight miRNAs and 31 mRNAs, most of which were all interconnected in a single cluster (Fig. 4A). A second network was then created to visualise the interactions between miRNAs that were downregulated at all three timepoints, plotted against mRNAs that were upregulated at all timepoints (Fig. 4B). This network highlighted miRNAs that are downregulated throughout disease and are likely unable to regulate their downstream targets, resulting in overexpression of those mRNAs. As a supplementary analysis, we also created miRNA–mRNA networks at each timepoint separately to visualise any changes during disease progression (Figs S3–S8). Inverse miRNA–mRNA relationships were identified at all three ages, with substantial overlap in network composition across timepoints. Networks linking upregulated miRNAs to downregulated mRNAs were consistently more extensive than those linking downregulated miRNAs to upregulated mRNAs, while both network types showed broader connectivity at 150 days. These findings suggest that a core putative miRNA-mediated regulatory programme is established by 90 days and persists during disease progression, with expansion of potential regulatory interactions at the late-symptomatic stage.

**Fig. 4.**
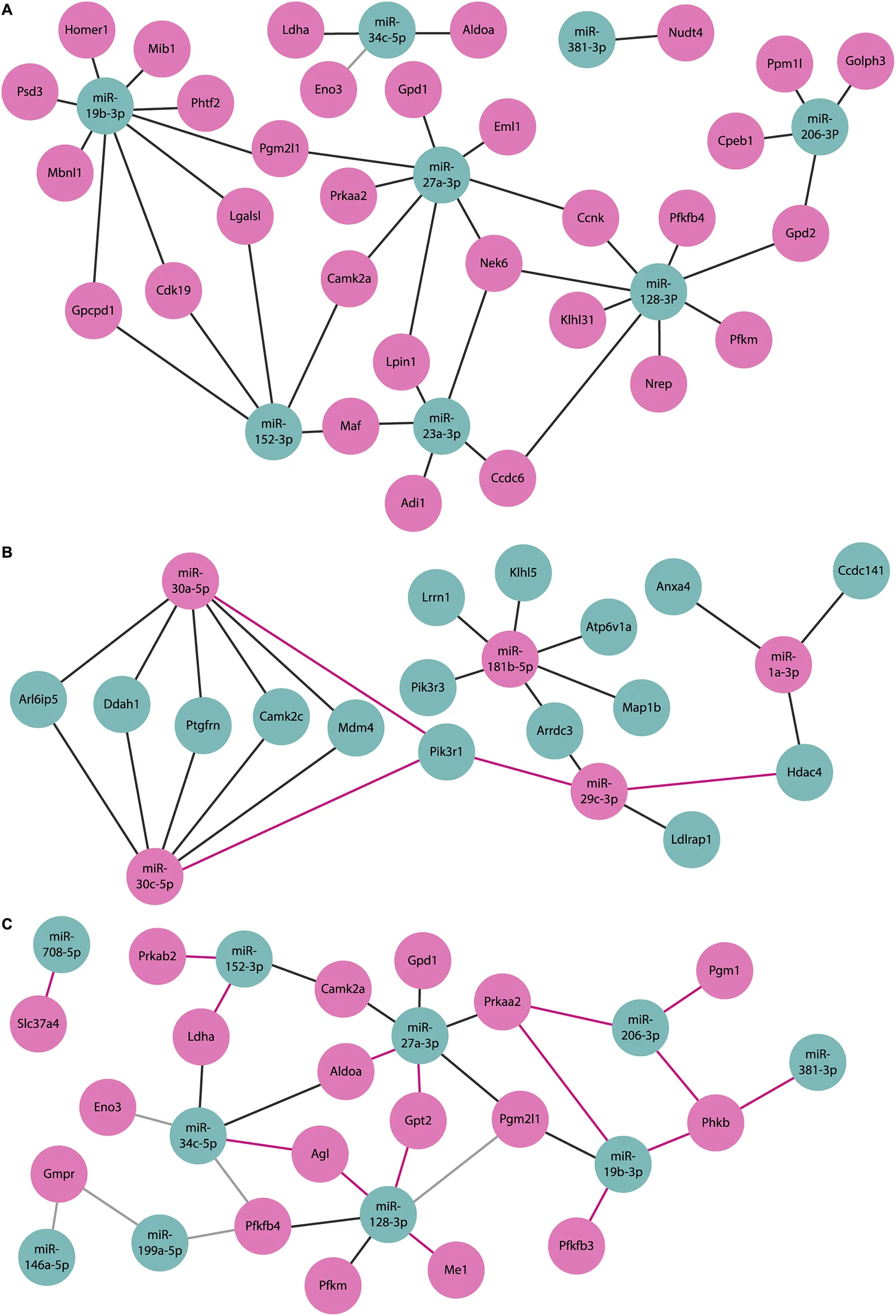
**Cytoscape analysis.** (A,B) Interactions between the upregulated miRNAs and downregulated mRNAs (A) or the downregulated miRNAs and upregulated mRNAs (B), at all three timepoints in the hSOD1^G93A^ muscle. (C) Network of miRNA–mRNA interactions enriched in carbohydrate metabolism pathways. Turquoise nodes represent upregulated miRNAs or mRNAs; pink nodes represent downregulated mRNAs or miRNAs. Grey connecting lines represent interactions from TargetScan, magenta lines represent interactions from TarBase, and black lines represent interactions from both.

Subsequently, the pathway enrichment analyses from the miRNA- and mRNA-sequencing data were used to create a more specific network related to the most enriched pathways. Of the genes identified to be downregulated at all timepoints in the hSOD1^G93A^ muscle, 37 genes appeared in the top four DAVID clusters related to carbohydrate metabolism. These genes were inputted into Cytoscape as the target nodes, then the reverse annotation information from the databases was used to plot all miRNAs interacting with these genes. The miRNAs were then filtered to include only those found to be upregulated in the muscle at all three timepoints, upon the assumption that overexpression of the miRNAs results in downregulation of the target mRNAs. The resulting network included a cluster of ten miRNAs and 18 of the input mRNAs, all interconnected by validated or predicted annotations (Fig. 4C). The miRNAs featured in this network likely have a direct influence on glucose metabolism and energy storage in the muscle. It is, therefore, possible that dysregulation of these miRNAs attributed to the hSOD1^G93A^ ALS-linked variant contribute to the suppression of glycolytic pathways in hSOD1^G93A^ muscle, a molecular pattern consistent with, and potentially contributing to, the fast-to-slow remodelling previously reported in SOD1 models.

### RT-qPCR confirms dysregulation of miRNAs in hSOD1^G93A^ mouse gastrocnemius muscle

To confirm the dysregulation of the most promising miRNAs and mRNAs identified in our RNA-sequencing (RNA-seq) analyses, we performed RT-qPCR on the sequenced samples and an additional two to four biological replicates per genotype and timepoint. We selected ten of the most promising miRNAs to assay when considering all expression, pathway enrichment and transcript interaction data. Of these, miR-1a-3p, miR-29c-3p, miR-30a-5p, miR-30c-5p, miR-100-5p and miR-181a-1-3p were significantly downregulated, whereas miR-21a-5p, miR-34c-5p, miR-206-3p and miR-362-5p were significantly upregulated, in the hSOD1^G93A^ muscle samples, compared to those in both control groups (main effect of genotype, *P*<0.0001), corroborating the RNA-seq findings (Fig. 5A–J). Additionally, miR-21a-5p, miR-29c-3p, miR-100-5p and miR-362-5p had a significant main effect of age (*P*=0.0307, *P*<0.0001, *P*=0.0002 and *P*<0.0001, respectively), and miR-29c-3p had a significant interaction between age and genotype (*P*=0.0129). For miR-21a-5p and miR-29c-3p, the age significance was lost following post-hoc comparisons. For miR-100-5p, post-hoc tests revealed an age-dependent increase in expression (Fig. 5E), particularly in the NTg group, whereas for miR-362-5p there was an age-dependent decrease in expression in all three genotypes (Fig. 5J).

**Fig. 5.**
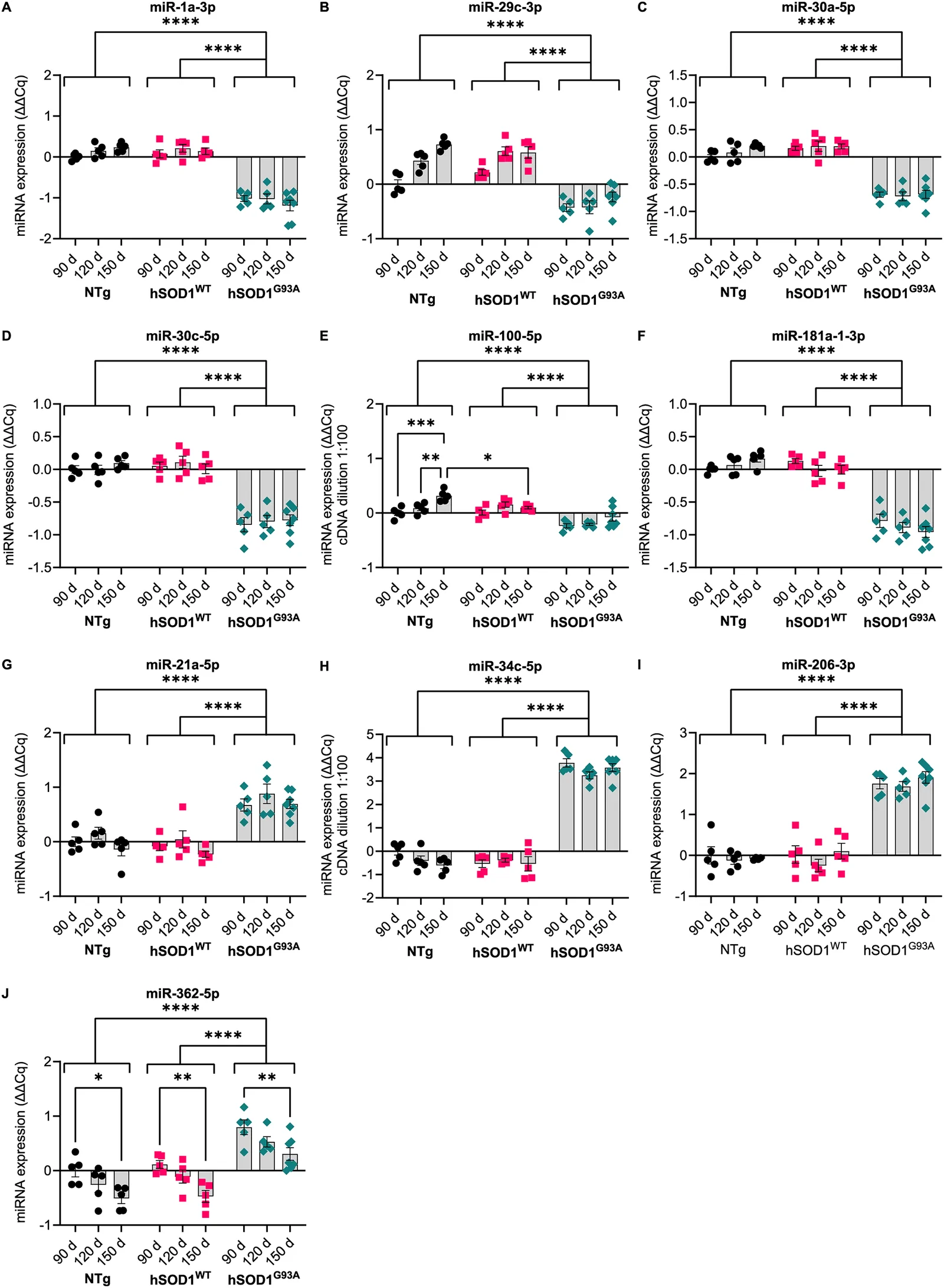
**Reverse transcription quantitative PCR (RT-qPCR) validation of dysregulated miRNAs in hSOD1^G93A^ muscle.** (A–F) Downregulated miRNAs. (G–J) Upregulated miRNAs. Following normalisation to the stable reference miRNA, miR-425-5p, expression values (ΔC_q_) were normalised to the mean of the 90-day NTg samples to obtain ΔΔC_q_ values [i.e. ΔΔC_q_ values were calculated as (90-day NTg ΔC_q_ mean)−(individual ΔC_q_ value)]. This normalisation process results in the 90-day NTg sample values being centred around zero. Data are shown as mean±s.d. and were analysed by a two-way ANOVA with Tukey's post-hoc. *N*=5–7 biological replicates per genotype per age. *P<0.05, **P<0.01, ***P<0.001, ****P<0.0001.

### RT-qPCR confirms dysregulation of mRNAs in hSOD1^G93A^ mouse gastrocnemius muscle

We selected 11 mRNAs to assay by RT-qPCR, predominantly those involved in the glycolysis pathway and some involved in broader metabolic and signalling pathways. Corroborating the RNA-seq data, phosphoglucomutase 1 (*Pgm1*), triosephosphate isomerase 1 (*Tpi1*), phosphoglycerate kinase 1 (*Pgk1*), enolase 3 (*Eno3*), pyruvate kinase muscle isozyme (*Pkm*), lactate dehydrogenase A (*Ldha*), fructose bisphosphate 2 (*Fbp2*) and protein kinase AMP-activated catalytic subunit alpha 2 (*Prkaa2*) were found to be significantly downregulated in the hSOD1^G93A^ muscle, compared to those in muscle of both control groups, at all timepoints (Fig. 6A–H)**.** In contrast, aldehyde dehydrogenase 2 family member (*Aldh2*), histone deacetylase 4 (*Hdac4*) and phosphatidylinositol 3-kinase regulatory subunit 1 (*Pik3r1*) were significantly upregulated in the hSOD1^G93A^ muscle, again corroborating the RNA-seq data (Fig. 6I–K)**.** All genes had a significant main effect of genotype (*P*<0.0001), with post-hoc comparisons revealing these to be between all age points of the hSOD1^G93A^ group, compared to those of the two control groups. *Pgm1*, *Tpi1*, *Pkm*, *Ldha* and *Hdac4* also had a significant main effect of age (*P*=0.0439, *P*=0.0073, *P*<0.0001, *P*=0.0105 and *P*=0.0163, respectively). Furthermore, *Ldha* showed a significant interaction between genotype and age (*P*=0.0117). In summary, all selected muscle mRNA candidates that were significantly dysregulated in the RNA-seq data were subsequently validated by RT-qPCR in a larger cohort of five to seven mice per genotype.

**Fig. 6.**
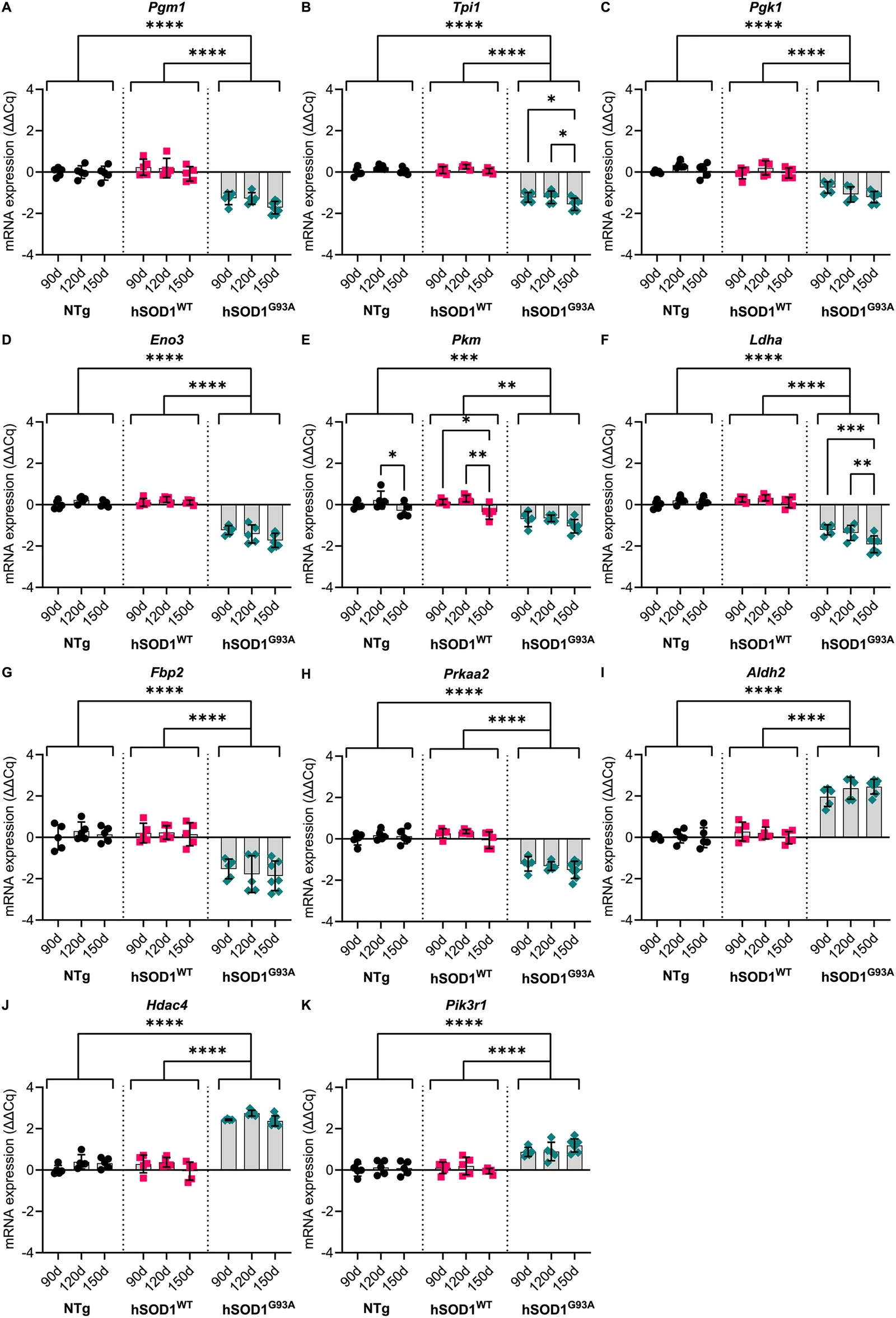
**RT-qPCR validation of dysregulated mRNAs in hSOD1^G93A^ muscle.** (A–H) Downregulated mRNAs. (I–K) Upregulated mRNAs. Following normalisation to the stable reference gene, *Tbp*, expression values (ΔC_q_) were normalised to the mean of the 90-day NTg samples to obtain ΔΔC_q_ values [i.e. ΔΔC_q_ values were calculated as (90-day NTg ΔC_q_ mean)−(individual ΔC_q_ value)]. This normalisation process results in the 90-day NTg sample values being centred around zero. Data are shown as mean±s.d. and were analysed by a two-way ANOVA with Tukey's post-hoc. *N*=5–7 biological replicates per genotype per age. *P<0.05, **P<0.01, ***P<0.001, ****P<0.0001.

### Inverse expression of interacting miRNAs and mRNAs

We then explored whether there were inverse expression relationships between any of the miRNAs and mRNAs that we examined by RT-qPCR, based on their previously validated or predicted interactions that we showed in Fig. 4. As summarised in Table 1, we confirmed inverse expression of all miRNA‒mRNA pairs in the hSOD1^G93A^ muscle for which we had available RT-qPCR data, apart from the relationship between miR-206-3p and *Hdac4*. This finding suggests that additional regulatory factors influence *Hdac4* expression in the muscle, potentially overriding the expected miR-206-3p-mediated repression. One such factor could be miR-1a-3p (Table 1), which is another known regulator of *Hdac4* (Li et al., 2014) and was the most highly expressed miRNA in our RNA-seq data (35% of total miRNA expression, on average), thus likely having a dominant effect over miR-206-3p. Overall, the remaining inverse expressions we observed suggest that the dysregulated miRNA profile of the hSOD1^G93A^ muscle contributes to the dysregulated mRNA profile.

**Table 1.** Opposite regulation of interacting miRNAs and mRNAs in the hSOD1^G93A^ gastrocnemius

miRNA name	miRNA dysregulation	mRNA name	mRNA dysregulation	Source of interaction data*	Inverse relation-ship by RT-qPCR?
miR-1a-3p	down	*Hdac4*	up	TarBase, TargetScan	yes
miR-29c-3p	down	*Hdac4*	up	TarBase	yes
miR-29c-3p	down	*Pik3r1*	up	TarBase	yes
miR-30a-5p	down	*Pik3r1*	up	TarBase	yes
miR-30c-5p	down	*Pik3r1*	up	TarBase	yes
miR-34c-5p	up	*Ldha*	down	TarBase, TargetScan	yes
miR-34c-5p	up	*Eno3*	down	TargetScan	yes
miR-206-3p	up	*Prkaa2*	down	TarBase	yes
miR-206-3p	up	*Pgm1*	down	TargetScan	yes
miR-206-3p	up	*Hdac4*	up	TarBase	no

*TarBase provides validated interactions, whereas TargetScan provides predicted interactions.

## DISCUSSION

In this study, we investigated the dysregulation of miRNA and mRNA networks in the gastrocnemius muscle of mouse models of ALS to elucidate their contribution to metabolic impairment. Using a combination of high-throughput RNA sequencing, bioinformatic analyses and RT-qPCR validation, we identified significant and sustained differences in both miRNA and mRNA expression in hSOD1^G93A^ mice compared to hSOD1^WT^ and NTg controls.

A key finding of our work was the extensive dysregulation of miRNAs in the hSOD1^G93A^ muscle, with 48 miRNAs significantly altered at early timepoints (90 and 120 days) and 64 dysregulated miRNAs at 150 days, compared to those in both control groups. Notably, miR-206-3p and miR-34c-5p were consistently the most upregulated across all timepoints, whereas miR-29c-5p and miR-181a-2-3p were the most downregulated. Parallel mRNA profiling revealed that more than 500 transcripts were dysregulated at each timepoint (specifically, 558 at 90 days, 544 at 120 days and 685 at 150 days), compared to those in both control groups, with a predominant trend toward downregulation, particularly in genes involved in glycolysis and carbohydrate metabolism. Functional enrichment analyses further implicated several signal transduction pathways in the metabolic reprogramming of the muscle. Interestingly, one of them was the FoxO signalling pathway, which activates genes that accelerate the degradation of muscle proteins and drive muscle atrophy (Sandri et al., 2004). Our network analyses underscored direct links between dysregulated miRNAs and metabolic genes, a relationship that was further validated by demonstrating their opposite regulation through RT-qPCR. Additionally, our timepoint-specific enrichment analyses further indicated that the principal metabolic and regulatory abnormalities were already present at 90 days and remained broadly consistent through 120 days and 150 days. This suggests that the molecular programme underlying hSOD1^G93A^ muscle pathology is established early, with disease progression characterised predominantly by persistence and late-stage broadening of metabolic, stress response and immune-related disturbances rather than the emergence of an entirely distinct transcriptional programme.

Furthermore, we showed that the excess RNA recovered from hSOD1^G93A^ muscle is unlikely to be explained by selective mature rRNA accumulation alone. Instead, our findings are more consistent with a broader increase in RNA content per unit of atrophic muscle tissue, although targeted analysis of pre-rRNA and ribosome-biogenesis markers would be required to determine whether ribosomal biogenesis is directly altered.

Our findings support the interpretation that the pathogenic hSOD1^G93A^ variant drives a complex reprogramming of post-transcriptional regulatory networks in skeletal muscle. The significant upregulation of miRNAs such as miR-206-3p, previously reported to play roles in muscle regeneration and NMJ repair (Williams et al., 2009; Liu et al., 2012), suggests an intrinsic attempt by the muscle to counteract denervation and atrophy. However, the concomitant downregulation of metabolic genes indicates that these compensatory mechanisms are insufficient, leading instead to an energy deficit and impaired glycolytic capacity (Palamiuc et al., 2015; Dupuis et al., 2003; Scaricamazza et al., 2020; Steyn et al., 2020). Our observation that the hSOD1^WT^ controls did not exhibit such changes reinforces the notion that these alterations are specifically linked to the pathological variant rather than to transgene overexpression.

In the context of existing literature, our results align with previous studies demonstrating miRNA dysregulation in ALS muscle. For example, prior work has highlighted the role of miR-206 in modulating neuromuscular repair (Valdez et al., 2014; Williams et al., 2009), while other studies have reported similar metabolic impairments in the fast-twitch muscles of ALS models (Dupuis et al., 2003; Palamiuc et al., 2015; Dobrowolny et al., 2018a). The present study extends these findings by providing a comprehensive analysis of both miRNA and mRNA expression, offering novel insights into how altered miRNA expression may directly suppress metabolic genes and exacerbate muscle atrophy. Integration of these miRNAs with their oppositely regulated gene targets revealed a coherent network that converges on carbohydrate-handling genes (e.g. *Ldha*, *Pgm1*, *Eno3*) and on master metabolic nodes such as AMPK and GLUT4. This provides a mechanistic bridge between previous biochemical work showing early glycolytic failure in fast-twitch fibres of SOD1 models (Dupuis et al., 2003; Palamiuc et al., 2015; Dobrowolny et al., 2018a; Scaricamazza et al., 2020; Steyn et al., 2020) and our own observation that glycolysis/gluconeogenesis, pentose-phosphate and related pathways are among the pathways most significantly enriched in downregulated genes. Our integrated miRNA–mRNA analysis suggests that mutant SOD1 muscle initiates an early miRNA-linked reprogramming of energy metabolism, which coincides with the activation of catabolic/atrophy pathways and with transcriptional signatures of a fast-to-slow fibre-type transition (Dobrowolny et al., 2018a; Scaricamazza et al., 2020). By unifying transcript-level, pathway and network evidence across three disease stages, this study extends earlier reports of isolated metabolic or miRNA defects (Palamiuc et al., 2015; Scaricamazza et al., 2020; Dobrowolny et al., 2018a; Si et al., 2018), and positions miRNA-guided metabolic circuits as promising entry points for interventions aimed at preserving muscle function in ALS (Scaricamazza et al., 2020; Palamiuc et al., 2015).

To place our muscle findings within the wider temporal pathology of the motor unit, it is important to note that the 90-day timepoint, although clinically presymptomatic in our colony, is unlikely to represent an absence of neural pathology. Although the precise timing varies between SOD1^G93A^ colonies, loss of fast-fatigable motor units and NMJ innervation has been reported well before overt symptoms, including in the medial gastrocnemius, while distal axon degeneration precedes or accompanies the onset of spinal MN loss (Chiu et al., 1995; Fischer et al., 2004; Hegedus et al., 2008). Therefore, the extensive miRNA and mRNA dysregulation detected in gastrocnemius at 90 days occurs during an established phase of motor unit remodelling, when vulnerable fast motor units are being lost and surviving MNs may undergo compensatory reinnervation (Schaefer et al., 2005). By 120 days, NMJ denervation, motor unit loss and spinal MN degeneration are expected to be advanced, while 150 days represents late-stage pathology across the motor unit. Notably, however, the muscle miRNA–mRNA signature was already largely established at 90 days and showed relatively little further change through 150 days. This relative stability contrasts with the progressive NMJ denervation, motor unit loss and spinal MN degeneration reported across the corresponding disease stages in SOD1^G93A^ mice (Fischer et al., 2004; Hegedus et al., 2008; Schaefer et al., 2005). This temporal pattern suggests that the muscle transcriptomic programme is an early, sustained component of disease rather than a molecular readout that simply increases in proportion to MN loss. It likely reflects a combination of muscle-intrinsic mutant SOD1 effects, denervation-induced remodelling and compensatory regenerative signalling, including the marked induction of miR-206. These processes may interact across the muscle–NMJ–MN axis; however, because NMJ and spinal cord pathology were not measured in our animals, their causal and temporal relationships cannot be determined directly from the present data.

Our study has several limitations. First, owing to the substantial costs and logistical challenges associated with maintaining the SOD1^G93A^ mouse colony, it was not feasible to include both male and female animals in this study. Consequently, our analyses were restricted to male mice; therefore, the potential influence of sex on the observed miRNA–mRNA expression patterns and interactions remains to be determined. Second, although our experimental design included two control groups to parse out variant-specific effects, the relatively modest sample sizes inherent to animal studies may limit the generalisability of our findings. Third, although our bioinformatic predictions of miRNA–mRNA interactions were supported by RT-qPCR validation, functional studies (e.g. miRNA mimic/inhibitor experiments) and protein expression analyses are needed to definitively establish causality. Fourth, our study focused on a specific time course in mouse models, and although these models recapitulate many aspects of ALS, they do not fully encompass the clinical and molecular heterogeneity observed in humans (Acevedo-Arozena et al., 2011). Nonetheless, by simultaneously profiling miRNAs and mRNAs across several timepoints, we were able to uncover robust, variant-specific changes in gene regulation that are linked to metabolic dysfunction. Moving forward, the dysregulated miRNAs and mRNAs identified in this mouse model should be validated in human ALS muscle samples using RT-qPCR to assess the translatability of these miRNA–mRNA interactions.

Finally, our analysis was restricted to the gastrocnemius and, therefore, cannot establish whether the identified miRNA–mRNA relationships are shared across muscles with different fibre-type compositions. Although the gastrocnemius is predominantly fast twitch (Sher and Cardasis, 1976), it contains spatially heterogeneous glycolytic and oxidative regions (Dimauro et al., 2019), and bulk RNA analysis of the entire muscle cannot attribute expression changes to an individual fibre type. Comparison with a predominantly fast muscle such as the tibialis anterior (Kammoun et al., 2014), and a more oxidative muscle such as the soleus (Kammoun et al., 2014), would help determine whether the identified miRNAs are associated specifically with the preferential vulnerability and fast-to-slow remodelling of glycolytic muscles. These muscles were not collected from the present animals, and such cross-muscle validation should therefore be undertaken in future studies. Nevertheless, previous studies provide evidence that the metabolic and fibre-type changes examined here are particularly prominent in faster muscles. Functional motor unit loss occurs earlier in the tibialis anterior, extensor digitorum longus and medial gastrocnemius than in the soleus of SOD1^G93A^ mice (Hegedus et al., 2007). In gastrocnemius, disease progression is accompanied by reduced type IIb myosin and increased type I and IIa/x myosin expression, consistent with a transition away from a fast-glycolytic phenotype (Capitanio et al., 2012). Similarly, muscle-restricted expression of SOD1^G93A^ produces increased oxidative staining, slow myosin expression and activation of a miR-499–MEF2C-associated slow-muscle programme in the tibialis anterior (Dobrowolny et al., 2018b). These findings support the interpretation that the metabolic transcript changes identified in the present study occur within a broader programme of remodelling in vulnerable, predominantly fast muscles.

In conclusion, our data indicate that the hSOD1^G93A^ variant induces widespread dysregulation of miRNA and mRNA expression in skeletal muscle, leading to impaired carbohydrate metabolism and energy homeostasis. These molecular alterations likely contribute to the fast-to-slow fibre-type switch and progressive muscle atrophy observed in ALS (Dobrowolny et al., 2018a; Scaricamazza et al., 2020). Understanding the interplay between miRNAs and their target mRNAs opens avenues for novel therapeutic strategies aimed at restoring metabolic balance in affected muscle tissue, which may, ultimately, help to slow disease progression and improve clinical outcomes in people with ALS.

## MATERIALS AND METHODS

### Animals

Procedures involving animals were approved by the UK Home Office under the UK Animals Scientific Procedures Act 1986 and the University of Sussex Animal Welfare and Ethics Review Board. Mice (*Mus musculus*) were kept on a 12 h light/dark cycle (lights on at 07:00) in cages with up to five littermates, and free access to food and water. Littermates were used for experiments wherever possible. Mice were obtained from The Jackson Laboratory. Male transgenic mice carrying the wild-type human *SOD1* gene (JAX#002297), or the human *SOD1* gene with the ALS-linked *G93A* variant (JAX#002726), were bred with female C57BL/6J mice (JAX#000664) to create in-house colonies. These mice are referred to as hSOD1^WT^ and hSOD1^G93A^, respectively. The lifespan of hSOD1^G93A^ mice is ∼130–150 days, whereas hSOD1^WT^ mice have a normal lifespan of ∼2 years (Gurney et al., 1994). C57BL/6J mice were used as non-transgenic (NTg) controls.

### Sample collection and RNA extraction

The gastrocnemius muscle was collected from five male hSOD1^G93A^ mice at the clinically pre-symptomatic (90 days old) and early-symptomatic (120 days old) timepoints, and from eight male hSOD1^G93A^ mice at the late-symptomatic (150 days old) timepoint. These ages were selected to capture the pathological transitions of the disease, from molecular onset to symptomatic progression and late-stage degeneration, allowing temporal characterisation of disease-associated molecular changes in skeletal muscle. Gastrocnemius tissue was also collected from five male hSOD1^WT^ and five male NTg mice at these timepoints, as age-matched controls. Mice were euthanised by terminal anaesthesia (intraperitoneal pentobarbital, 200 mg kg^−1^) and the gastrocnemius muscle was collected from both hindlimbs. The tissue samples were snap frozen in liquid nitrogen and transferred to −80°C for long-term storage.

Each mouse was randomly assigned either the left or right hindlimb gastrocnemius muscle from which to extract RNA. For the hSOD1^G93A^ mice, total RNA was extracted from the entire muscle using an miRNeasy Mini Kit (Qiagen, 217084), as per the manufacturer's guidelines, with the following modifications. To account for the variation in muscle size due to muscle atrophy in the hSOD1^G93A^ mice, the entire muscle was lysed. To do so, up to 100 mg of tissue was loaded into 2 ml homogenising tubes containing 1.4 mm ceramic beads (Precellys, CK14) and lysed in 1 ml Qiazol. Muscles exceeding 100 mg in mass were split into two homogenisation tubes. Following lysis and phase separation, the aqueous phase containing RNA was pooled from both homogenisation tubes. To then prevent overloading of the RNA extraction column, which has a capacity for up to 50 mg of starting material, a proportional volume of the aqueous phase, equivalent to 30 mg of input muscle, was aliquoted for downstream RNA extraction. For example, from a 150 mg tissue lysed and processed to give a total aqueous phase volume of 1 ml, a 200 μl aliquot of the aqueous phase was used for downstream RNA extraction, representing a proportion equivalent to 30 mg. As the aqueous phase contained a homogenous mix of total RNA from the entire muscle, the aliquot taken from each sample was representative of the total RNA pool expressed in the tissue. The amount of RNA extracted from each muscle was then quantified using a Qubit™ RNA high sensitivity assay kit (Fisher Scientific, 10034622) with the Qubit™ 3.0 fluorometer.

### Library preparation and RNA sequencing

A QIAseq miRNA Library Kit (Qiagen, 331505) was used with 100 ng total RNA to prepare miRNA libraries for next-generation sequencing, according to the manufacturer's protocol. All bead-washing steps were performed using 500 μl of 80% ethanol instead of the recommended 200 μl. The plate indices from a QIAseq miRNA NGS 96 Index IL (Qiagen, 331565) were used for library preparation. The QuantSeq 3′ mRNA-Seq Single Indexing Library Prep Kit for Illumina (FWD) (Lexogen, 015) was used with 500 ng total RNA to prepare mRNA libraries for next-generation sequencing, as per the manufacturer's protocol. During the PCR amplification step, i7 indexing primers were introduced to the 5′ end of complementary DNA (cDNA) libraries to enable multiplexing.

Pre-sequencing quality control of the miRNA and mRNA libraries was performed using a High Sensitivity D1000 ScreenTape (Agilent, 5067-5584) and High Sensitivity D1000 Reagents (Agilent, 5067-5585), on an Agilent TapeStation instrument. Then, a Qubit™ dsDNA High Sensitivity Assay Kit (Invitrogen™, Q32851) was used with the Qubit™ 3.0 fluorometer to quantify the amount of library before diluting and pooling the samples at equimolar concentrations for sequencing.

Sequencing of the miRNA libraries was performed by the Next Generation Sequencing Facility at the University of Leeds, where a BluePippin (Sage Science) was used to size select for the libraries between 160 and 200 bp, to extract miRNA libraries. The libraries were then prepared for 100 bp single-end sequencing on an Illumina NextSeq2000 100 bp single-end P2 flow cell. Upon completion of the sequencing, the sequencing facility performed basecalling, demultiplexing and trimmed the 5′ adapters from the reads. Sequencing of the mRNA libraries was performed by the next-generation sequencing service provided by BGI Genomics, on a DNBSEQ-g400-FLC flow cell (MGI Tech) by 100 bp single-end sequencing. Upon completion of the sequencing, the sequencing facility then performed the basecalling and demultiplexing.

### Bioinformatic analysis

Bioinformatic analysis of the miRNA- and mRNA-sequencing data was performed separately, using open-sourced script packages via the Galaxy webserver (The Galaxy Community, 2024). Quality control of the sequencing data was undertaken using the FastQC tool, and reads were trimmed using Cutadapt (Martin, 2011). For miRNA reads, the 3′ adapter was removed, and any reads without adapters or outside of the size range of 17-60 bp were removed. For mRNA reads, the specific 3′ adapter sequence (5′-A{18}AGATCGGAAGAGCACACGTCTGAACTCCAGTCAC-3′) was removed, as were the long poly-A tails on the reads, which were removed by trimming an artificial adapter sequence of A{100}. In addition, as per the library synthesis kit recommendations, an additional 12 bases were trimmed from the 5′ end of the reads owing to the higher potential of mismatches in this region caused by the random priming during library synthesis.

miRNA reads were subsequently aligned to the latest mouse genome assembly available at the time of analysis (GRCm38.p6; RefSeq assembly accession GCF_000001635.26), using Bowtie2, with the default ‘sensitive end-to-end’ settings (Langmead and Salzberg, 2012). Reads that aligned to the mouse genome were then mapped to miRNAs in the same genome, obtained from the miRBase database of miRNA sequences (Kozomara et al., 2019), and quantified using featureCounts (Liao et al., 2014). Separately, mRNA reads were aligned to the same genome using the Hisat2 alignment package with default settings (Kim et al., 2019). Then, featureCounts was used to annotate reads to the Ensembl annotation gtf release 102, which is based on the GRCm38 genome and, hence, corresponds to the same assembly that the reads were aligned to.

Secondary analysis to calculate differential miRNA and mRNA expression between the different samples was undertaken using the DESeq2 package (Love et al., 2014) via the Galaxy webserver. We designed our analysis to enable comparison of genotype effects at each timepoint, and timepoint effects within each genotype. As such, all samples within one genotype and timepoint group were compared to all other genotype and timepoint groups. This approach was chosen to allow focused, genotype-specific exploration of timepoint effects, avoiding the assumption that changes over time would be uniform across genotypes.

From this, the relevant group comparisons were selected for downstream wider comparison. All genotypes were compared at each individual timepoint; then, within each genotype, expression was compared at different timepoints. For miRNA differential expression analysis, differences were considered biologically significant when the following criteria were met: average miRNA expression exceeded 100 reads per sample; log_2_fold change was equal to or exceeding ±1.5; adjusted *P*-value was ≤0.05. For mRNA differential expression analysis, the significance threshold was more stringent than for miRNAs to reduce the chance of false positives, given the increased number of mRNAs (∼23,000 mRNAs, versus only ∼1000 miRNAs). Expression differences were considered biologically significant when the following criteria were met: average mRNA expression exceeded 200 reads per sample, log_2_fold change was equal to or exceeding ±2; adjusted *P*-value was ≤0.01.

A variety of different tools were used to explore the predicted and confirmed gene targets of miRNAs identified to be significantly dysregulated in these studies. DIANA-MiRPath (version 4.0) (Tastsoglou et al., 2023) was used to identify experimentally validated miRNA targets from the Tarbase (version 8) (Karagkouni et al., 2018) and miRTarBase (version 9) (Huang et al., 2022) databases and predicted conserved targets from TargetScan (version 8) (McGeary et al., 2019). MiRWalk (Sticht et al., 2018) was used to identify predicted miRNA targets from the unique TarPmiR prediction algorithm (Ding et al., 2016). Finally, miRSystem (Lu et al., 2012) was also used, which integrates seven miRNA target gene prediction algorithms (DIANA, miRanda, miRBridge, PicTar, PITA, rna22 and TargetScan) and two experimentally validated databases (TarBase and miRecords), to identify targets. Pathways with a *P*-value <0.05 were considered significant.

When analysing a pre-defined group of genes for functional enrichment, the DAVID bioinformatics resource system was used (Sherman et al., 2022). Pathways with false discovery rates (FDRs) <0.05 were considered to be significantly enriched, and gene clusters with enrichment scores equivalent of *P*<0.05 (enrichment score >1.33) were considered statistically significant.

To explore disease relevant miRNA–mRNA interactions, visual networks of dysregulated miRNAs and mRNAs were created using the CyTargetLinker app (Kutmon et al., 2018) in Cytoscape (Shannon et al., 2003). Networks were created using annotation data from both validated miRNA–gene interactions from TarBase and also predicted miRNA–gene interactions from TargetScan. It is important to acknowledge that miRNA–mRNA interactions are complex, and although an interaction may be validated, there may be more influential interactions that determine overall expression of a particular miRNA or gene. Therefore, to increase the identification of real disease-relevant interactions, only miRNA and mRNA interactions whereby the expressions were changed in opposing directions were explored (i.e. upregulated miRNA and downregulated mRNA or vice versa).

### RT-qPCR for mRNA

cDNA synthesis using 1600 ng total RNA was carried out using a High-Capacity RNA-to-cDNA kit (Thermo Fisher Scientific, 4388950), according to the manufacturer's protocol. A 50-fold dilution of the cDNA was then used for SYBR-Green RT-qPCR to quantify the expression levels of target mRNAs (normalised to the expression levels of the stably expressed reference gene *Tbp*), using SYBR qPCR 2× mix (AptoGen, 412200.5) and the primers listed in Table S2^0^.

### RT-qPCR for miRNAs

A total of 10 ng muscle RNA was used to synthesise cDNA of miRNAs with an TaqMan™ Advanced miRNA cDNA Synthesis Kit (Thermo Fisher Scientific, A28007), as per the manufacturer's guidelines. Following normalisation of miRNA expression with DESeq2, the miRNAs with the smallest standard deviation between the sequenced samples were identified, and the least variable miRNAs were selected as normaliser candidates for RT-qPCR validation. Specific miRNAs of interest were then used with TaqMan™ Advanced miRNA assays (Fisher Scientific, 15412184) to validate the sequencing results by RT-qPCR, using fast cycling, and with the qPCR Lo-ROX 2X mix (AptoGen, 412101.5). The manufacturer's protocol was followed for all miRNAs; however, the annealing temperatures of the miRNA assays were optimised using standard curves, to ensure qPCR efficiency between 90% and 110% for each miRNA assay. Some miRNA assays required a 100-fold dilution, as determined by the standard curves. All miRNA assays were normalised to the expression of mmu-miR-425-5p (mmu481161_mir), at either the 1:10 or 1:100 dilution of cDNA, depending on which dilution was used for that particular miRNA assay of interest. The TaqMan™ Advanced miRNA assays used for the target miRNAs were as follows: miR-1a-3p (mmu482914_mir), miR-29c-3p (mmu479229_mir), miR-30a-5p (479448_mir), miR-30c-5p (478008_mir), miR-100-5p (mmu478224_mir), miR-181a-3p (479405_mir), miR-21-5p (477975_mir), miR-34c-5p (478052_mir), miR-206-3p (477968_mir) and miR-362-5p (mmu481128_mir).

### Statistical analysis

RT-qPCR data were analysed in GraphPad Prism version 10.0.0. Outliers were identified by the ROUT test with *Q*=1% and were removed before further analysis. Data were analysed for normality using the Shapiro–Wilk test. Statistical comparisons between groups were conducted using a two-way analysis of variance (ANOVA) with Tukey's post-hoc test. Relationships between variables were analysed using Pearson's correlation tests. The threshold for significance was set to 5%.

## Supplementary Material

10.1242/dmm.052963_sup1 Supplementary information

Table S1. signficantly dysregulated miRNAs in hSOD1G93A muscle, compared to hSOD1WT muscle, at each timepoint.

Table S2. signficantly dysregulated miRNAs in hSOD1G93A muscle, compared to NTg muscle, at each timepoint.

Table S3. signficantly dysregulated mRNAs in hSOD1G93A muscle, compared to hSOD1WT muscle, at each timepoint.

Table S4. signficantly dysregulated mRNAs in hSOD1G93A muscle, compared to NTg muscle, at each timepoint.

Table S5. signficantly dysregulated miRNAs at 150d, compared to 90d, for each genotype.

Table S6. signficantly dysregulated mRNAs at 150d, compared to 90d, for each genotype.

Table S7. Enriched KEGG pathways of mRNAs upregulated in hSDO1G93A muscle at 90 days old.

Table S8. Enriched KEGG pathways of mRNAs downregulated in hSDO1G93A muscle at 90 days old.

Table S9. Enriched KEGG pathways of mRNAs downregulated in hSDO1G93A muscle at 120 days old.

Table S10. Enriched KEGG pathways of mRNAs downregulated in hSDO1G93A muscle at 120 days old.

Table S11. Enriched KEGG pathways of mRNAs downregulated in hSDO1G93A muscle at 150 days old.

Table S12. Enriched KEGG pathways of mRNAs downregulated in hSDO1G93A muscle at 150 days old.

Table S13. Enriched pathways of genes targeted by dysregulated miRNAs in hSOD1G93A muscle at 90 days old from the miRPath database

Table S14. Enriched pathways of genes targeted by dysregulated miRNAs in hSOD1G93A muscle at 120 days old from the miRPath database

Table S15. Enriched pathways of genes targeted by dysregulated miRNAs in hSOD1G93A muscle at 150 days old from the miRPath database

Table S16. Enriched pathways of genes targeted by dysregulated miRNAs in hSOD1G93A muscle at each age from the miRSystem database

Table S17. Enriched pathways of genes targeted by dysregulated miRNAs in hSOD1G93A muscle at 90 days old from the miRWalk database

Table S18. Enriched pathways of genes targeted by dysregulated miRNAs in hSOD1G93A muscle at 120 days old from the miRWalk database

Table S19. Enriched pathways of genes targeted by dysregulated miRNAs in hSOD1G93A muscle at 150 days old from the miRWalk database
