# Supplementary material for: Dysregulated microRNA–mRNA networks in SOD1G93A skeletal muscle reveal metabolic impairments in amyotrophic lateral sclerosis
Source: Dis Model Mech. 2026 Oct 9;19(10):dmm052963. doi: 10.1242/dmm.052963 (PMC13714871; doi:10.1242/dmm.052963)
Supplement: Supplementary file 1 [file dmm-19-052963-s1.pdf]

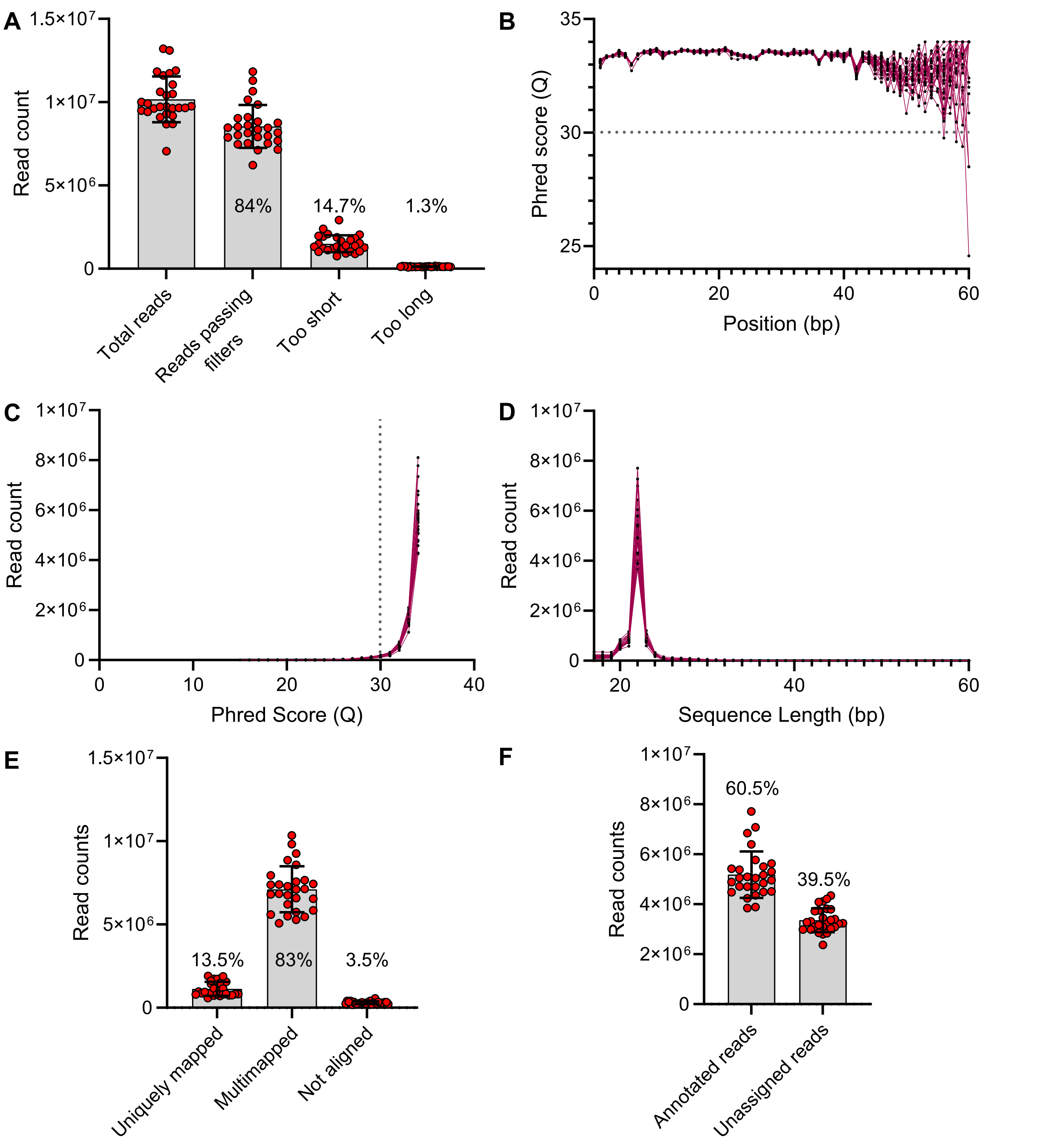

Fig. S1. Quality control of miRNA sequencing reads and mapping information. (**A**) Number of reads discarded and retained in the data after adapter trimming and filtering based on length. (**B**) Per-base sequence quality. (**C**) Per-sequence quality. (**D**) Sequence length distribution. (**E**) Number of mapped and unmapped reads in sequenced samples. (**F**) Number of reads that annotated to known miRNA sequences from miRBase. Data in **A**, **E** and **F** are shown as mean ± s.e.m. Data in **B**, **C**, and **D** are shown as mean. N = 27 samples.

Disease Models & Mechanisms • Supplementary information

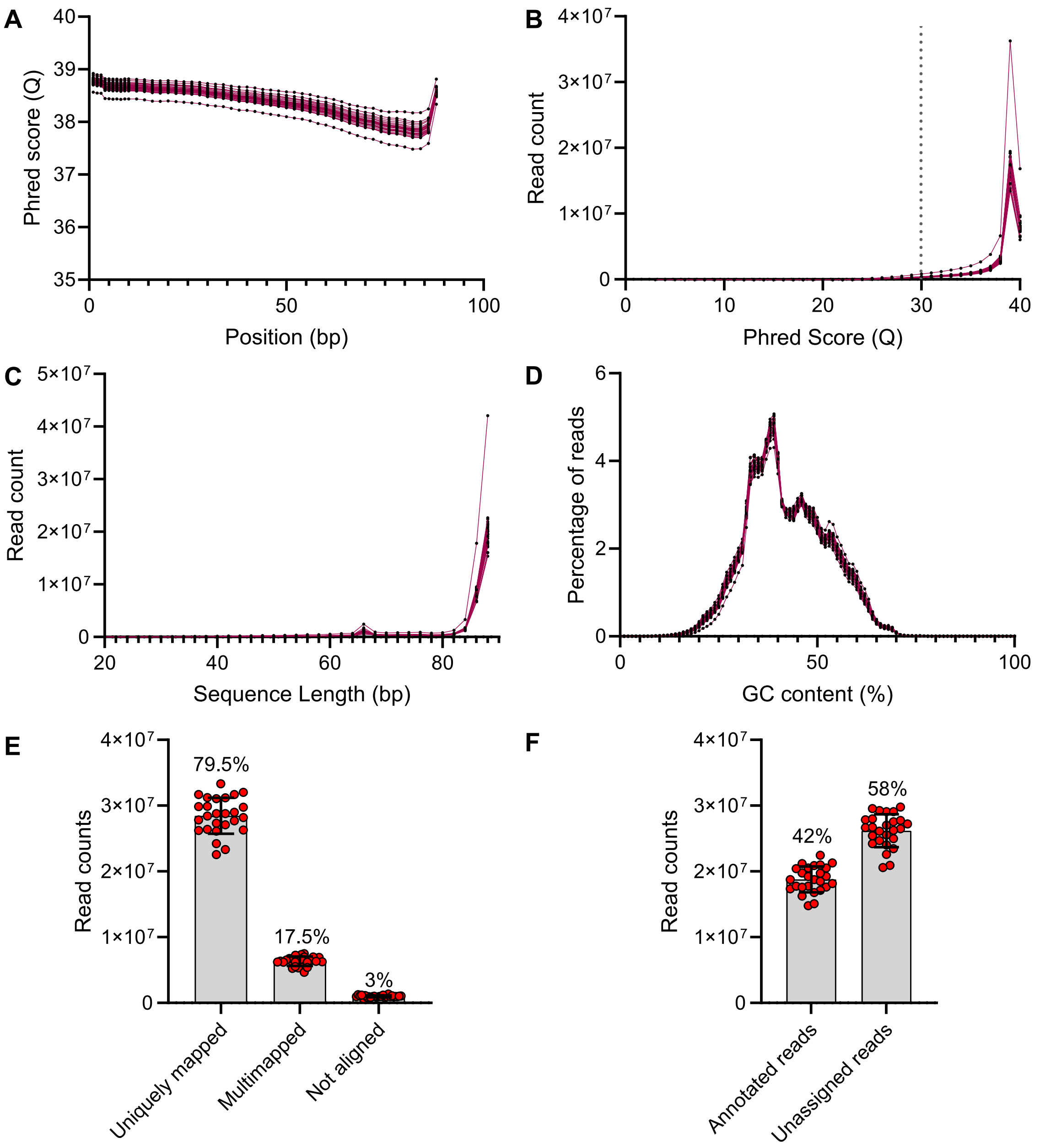

Fig. S2. Quality control of mRNA sequencing reads and mapping information. (**A**) Per-base sequence quality. (**B**) Per-sequence quality. (**D**) Sequence length distribution. (**D**) GC content. (**E**) Number of reads that aligned to the mouse genome uniquely, multiple times, or not at all. (**F**) Number of reads that annotated to known mRNA sequences from Ensembl. Data in **E** and **F** are shown as mean ± s.e.m. Data in **A-D** are shown as mean. N = 27 samples for **A-D**, and N = 26 samples for **E** and **F**.

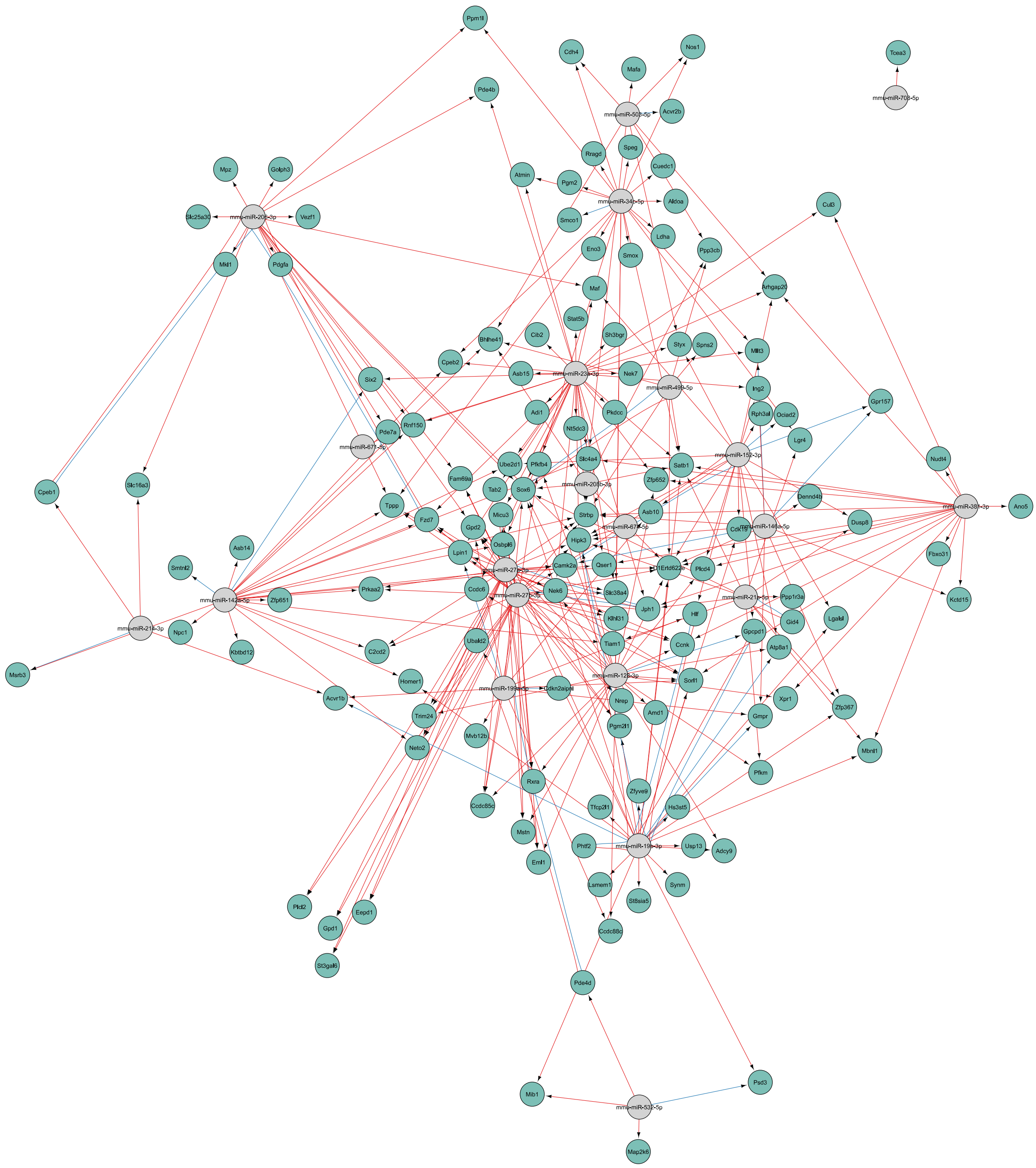

Fig. S3. Cytoscape analysis showing interactions between the upregulated miRNAs and downregulated mRNAs at 90 days old in the hSOD1^{G93A} muscle. Grey nodes represent upregulated miRNAs, and turquoise nodes represent downregulated miRNAs. Red connecting lines represent interactions from TargetScan and blue lines from TarBase.

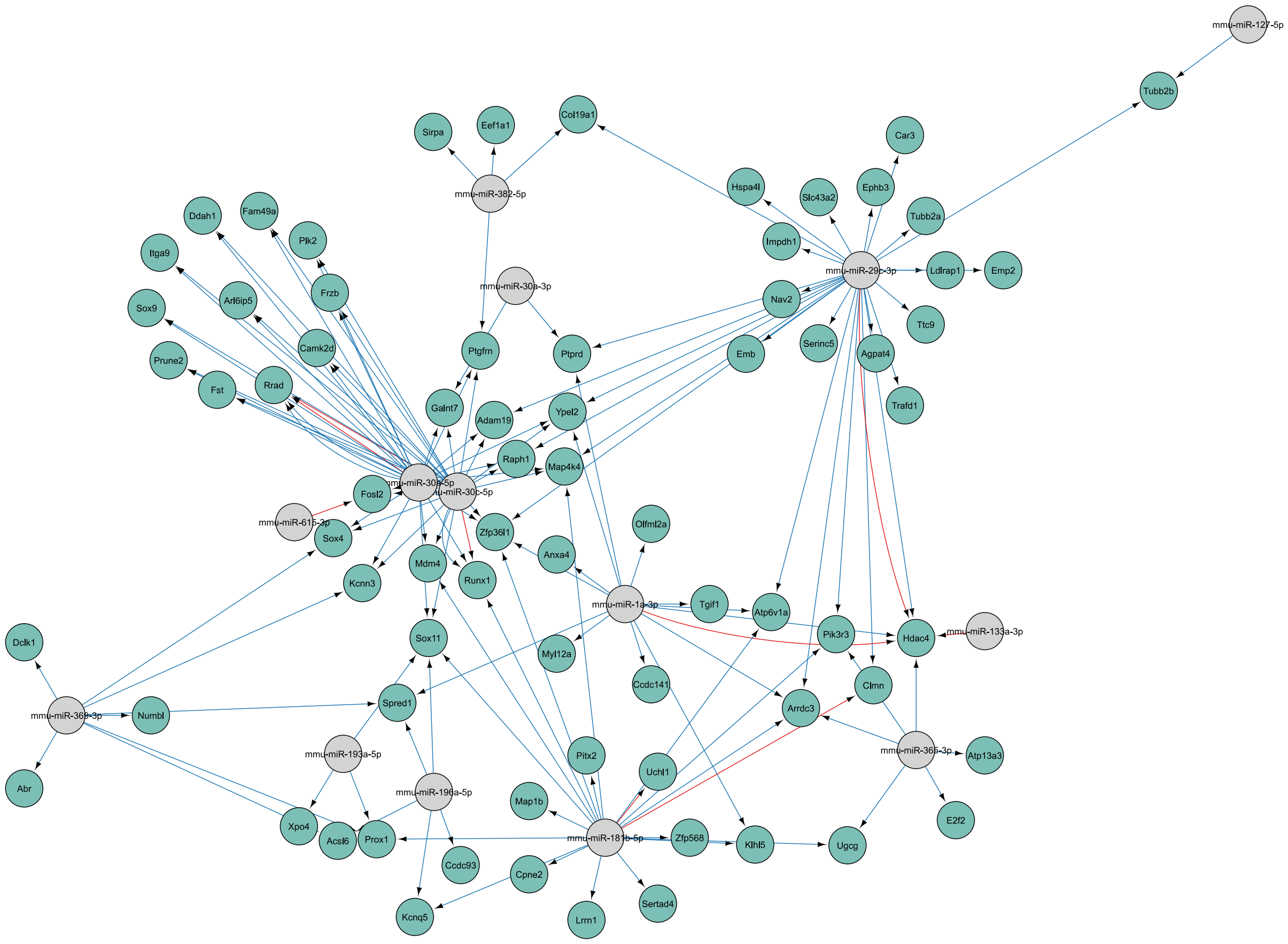

Fig. S4. Cytoscape analysis showing interactions between the downregulated miRNAs and upregulated mRNAs at 90 days old in the hSOD1^{G93A} muscle. Grey nodes represent downregulated miRNAs, and turquoise nodes represent upregulated miRNAs. Red connecting lines represent interactions from TargetScan and blue lines from TarBase.

Disease Models & Mechanisms • Supplementary information

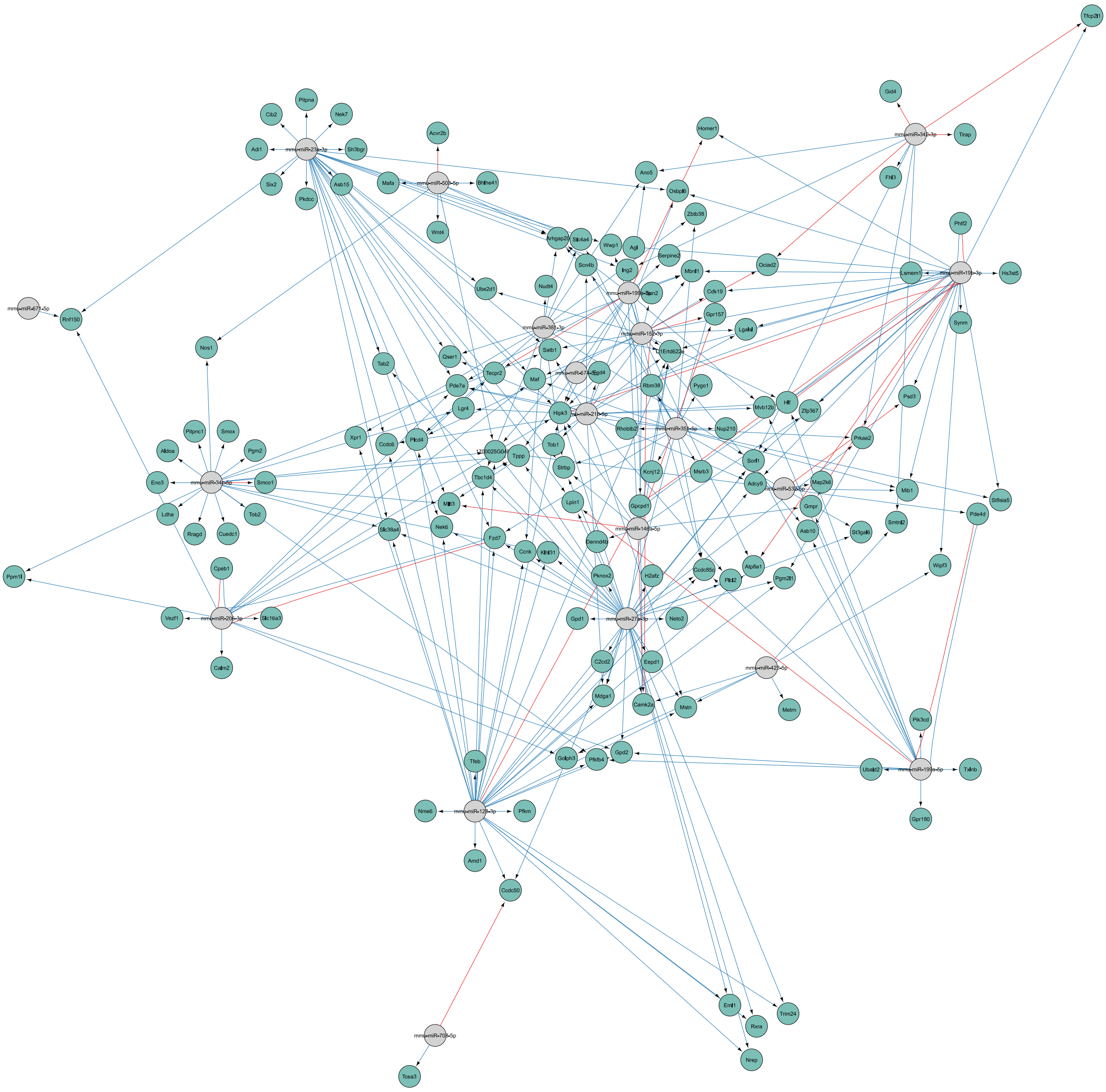

Fig. S5. Cytoscape analysis showing interactions between the upregulated miRNAs and downregulated mRNAs at 120 days old in the hSOD1^{G93A} muscle. Grey nodes represent upregulated miRNAs, and turquoise nodes represent downregulated miRNAs. Red connecting lines represent interactions from TargetScan and blue lines from TarBase.

Disease Models & Mechanisms • Supplementary information

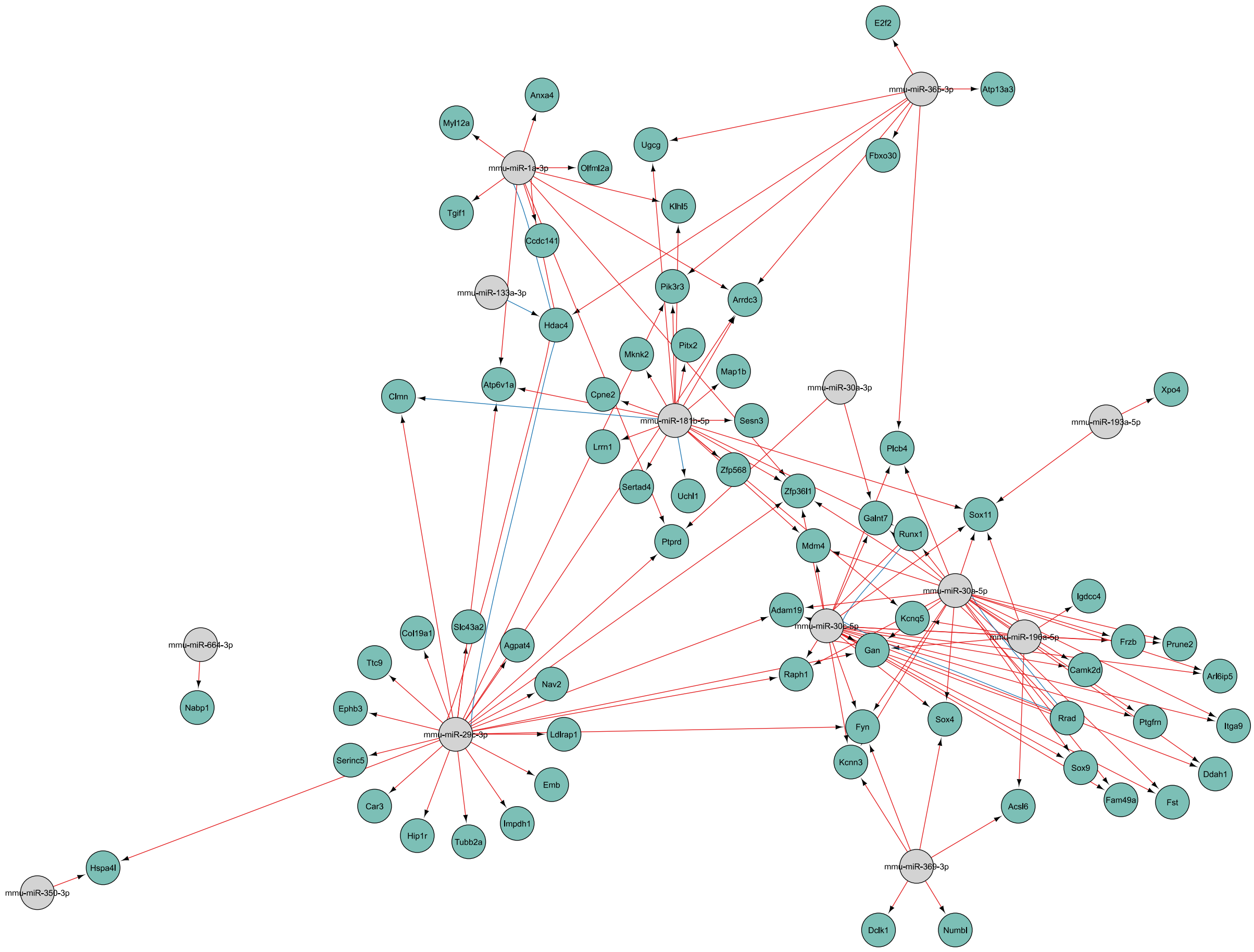

Fig. S6. Cytoscape analysis showing interactions between the downregulated miRNAs and upregulated mRNAs at 120 days old in the hSOD1^{G93A} muscle. Grey nodes represent downregulated miRNAs, and turquoise nodes represent upregulated miRNAs. Red connecting lines represent interactions from TargetScan and blue lines from TarBase.

Disease Models & Mechanisms • Supplementary information

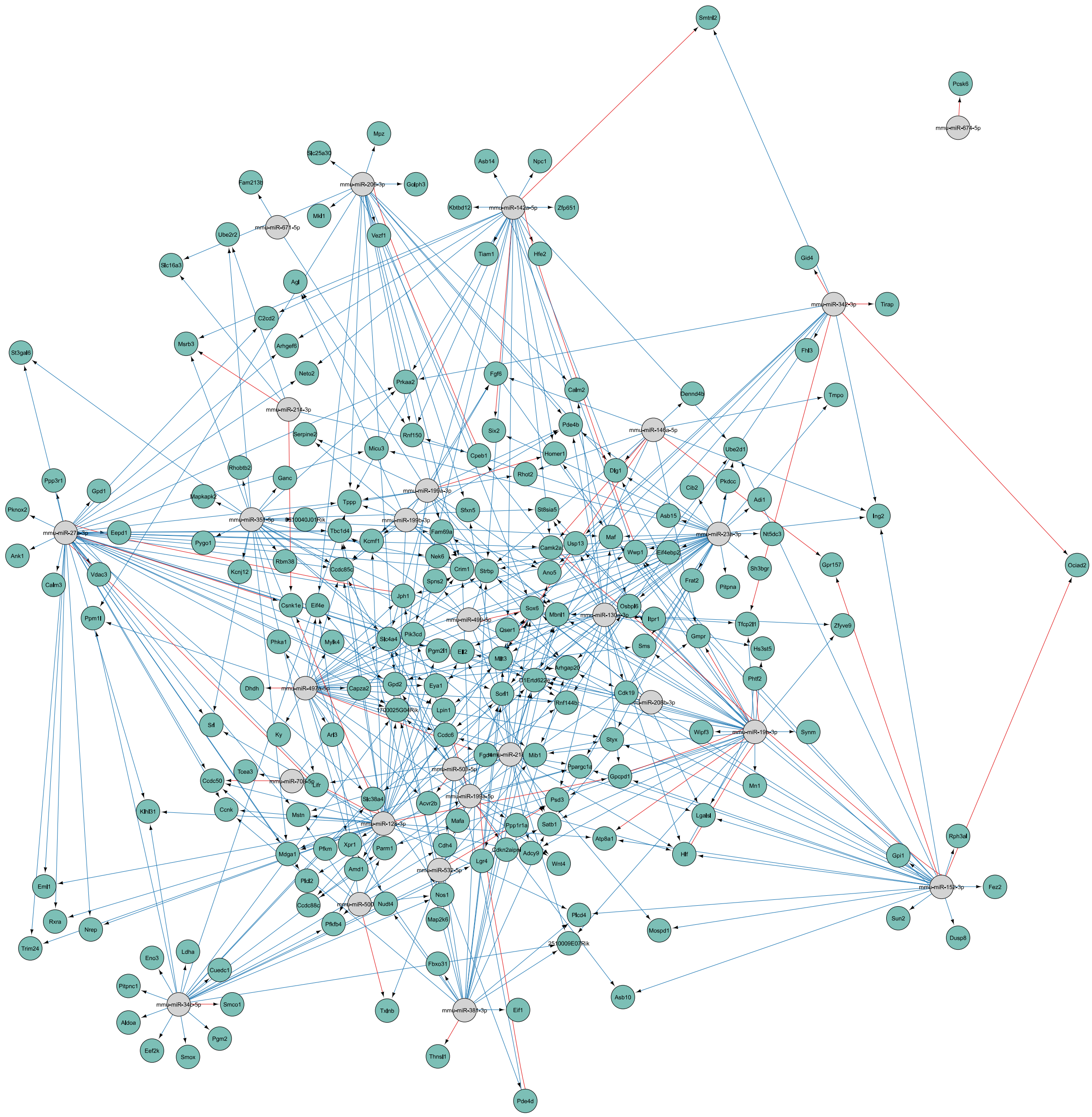

Fig. S7. Cytoscape analysis showing interactions between the upregulated miRNAs and downregulated mRNAs at 150 days old in the hSOD1^{G93A} muscle. Grey nodes represent upregulated miRNAs, and turquoise nodes represent downregulated miRNAs. Red connecting lines represent interactions from TargetScan and blue lines from TarBase.

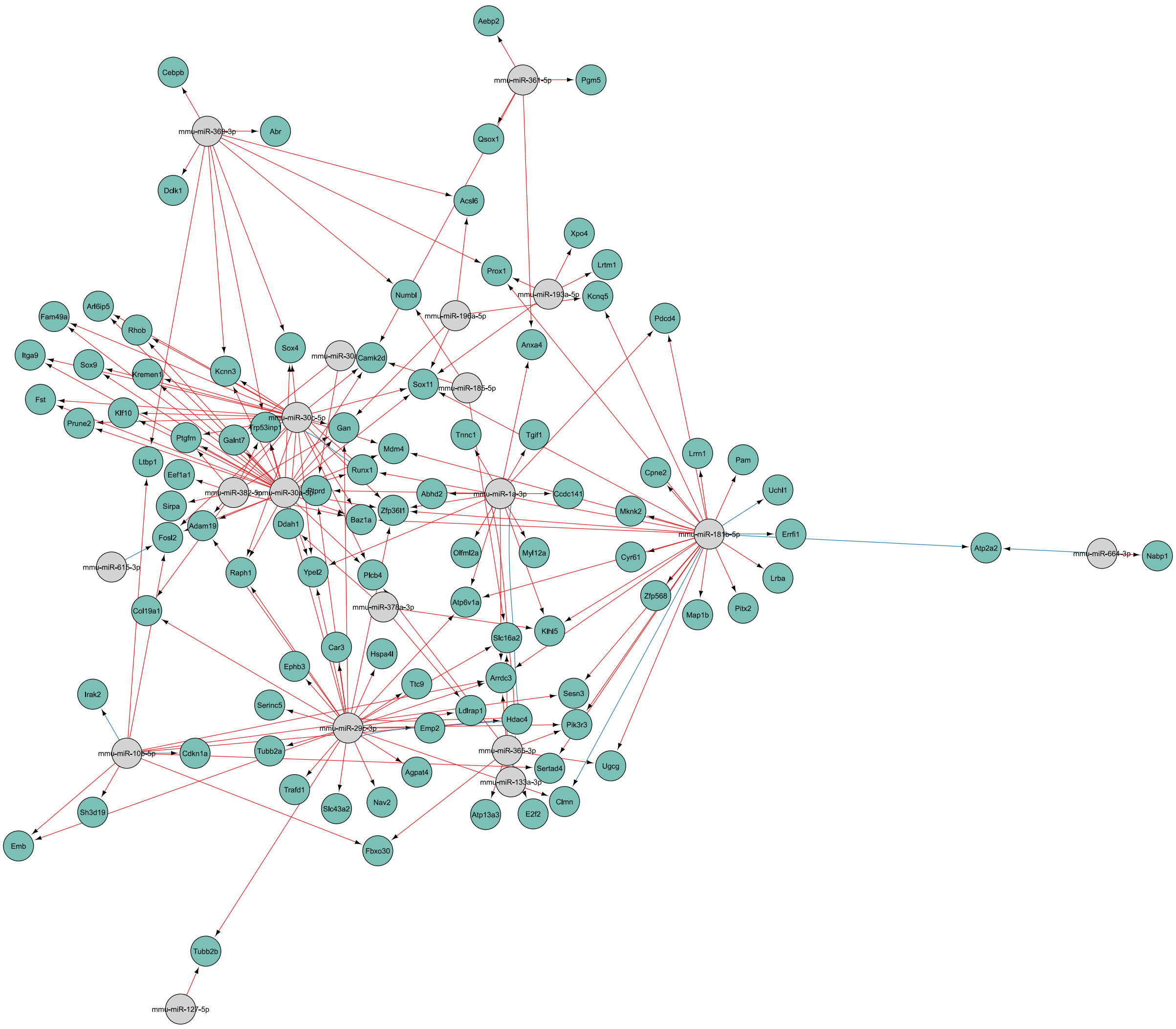

Fig. S8. Cytoscape analysis showing interactions between the downregulated miRNAs and upregulated mRNAs at 150 days old in the hSOD1[G93A] muscle. Grey nodes represent downregulated miRNAs, and turquoise nodes represent upregulated miRNAs. Red connecting lines represent interactions from TargetScan and blue lines from TarBase.

Disease Models & Mechanisms • Supplementary information

Table S1. signficantly dysregulated miRNAs in hSOD1G93A muscle, compared to hSOD1WT muscle, at each timepoint.

Available for download at
https://journals.biologists.com/dmm/article-lookup/doi/10.1242/dmm.052963#supplementary-data

Table S2. signficantly dysregulated miRNAs in hSOD1G93A muscle, compared to NTg muscle, at each timepoint.

Available for download at
https://journals.biologists.com/dmm/article-lookup/doi/10.1242/dmm.052963#supplementary-data

Table S3. signficantly dysregulated mRNAs in hSOD1G93A muscle, compared to hSOD1WT muscle, at each timepoint.

Available for download at
https://journals.biologists.com/dmm/article-lookup/doi/10.1242/dmm.052963#supplementary-data

Table S4. signficantly dysregulated mRNAs in hSOD1G93A muscle, compared to NTg muscle, at each timepoint.

Available for download at
https://journals.biologists.com/dmm/article-lookup/doi/10.1242/dmm.052963#supplementary-data

Table S5. signficantly dysregulated miRNAs at 150d, compared to 90d, for each genotype.

Available for download at
https://journals.biologists.com/dmm/article-lookup/doi/10.1242/dmm.052963#supplementary-data

Table S6. signficantly dysregulated mRNAs at 150d, compared to 90d, for each genotype.

Available for download at
https://journals.biologists.com/dmm/article-lookup/doi/10.1242/dmm.052963#supplementary-data

Table S7. Enriched KEGG pathways of mRNAs upregulated in hSDO1G93A muscle at 90 days old.

Available for download at
https://journals.biologists.com/dmm/article-lookup/doi/10.1242/dmm.052963#supplementary-data

Table S8. Enriched KEGG pathways of mRNAs downregulated in hSDO1G93A muscle at 90 days old.

Available for download at
https://journals.biologists.com/dmm/article-lookup/doi/10.1242/dmm.052963#supplementary-data

Table S9. Enriched KEGG pathways of mRNAs downregulated in hSDO1G93A muscle at 120 days old.

Available for download at
https://journals.biologists.com/dmm/article-lookup/doi/10.1242/dmm.052963#supplementary-data

Table S10. Enriched KEGG pathways of mRNAs downregulated in hSDO1G93A muscle at 120 days old.

Available for download at
https://journals.biologists.com/dmm/article-lookup/doi/10.1242/dmm.052963#supplementary-data

Table S11. Enriched KEGG pathways of mRNAs downregulated in hSDO1G93A muscle at 150 days old.

Available for download at
https://journals.biologists.com/dmm/article-lookup/doi/10.1242/dmm.052963#supplementary-data

Table S12. Enriched KEGG pathways of mRNAs downregulated in hSDO1G93A muscle at 150 days old.

Available for download at
https://journals.biologists.com/dmm/article-lookup/doi/10.1242/dmm.052963#supplementary-data

Table S13. Enriched pathways of genes targeted by dysregulated miRNAs in hSOD1G93A muscle at 90 days old from the miRPath database

Available for download at
https://journals.biologists.com/dmm/article-lookup/doi/10.1242/dmm.052963#supplementary-data

Table S14. Enriched pathways of genes targeted by dysregulated miRNAs in hSOD1G93A muscle at 120 days old from the miRPath database

Available for download at
https://journals.biologists.com/dmm/article-lookup/doi/10.1242/dmm.052963#supplementary-data

Table S15. Enriched pathways of genes targeted by dysregulated miRNAs in hSOD1G93A muscle at 150 days old from the miRPath database

Available for download at
https://journals.biologists.com/dmm/article-lookup/doi/10.1242/dmm.052963#supplementary-data

Disease Models & Mechanisms • Supplementary information

Table S16. Enriched pathways of genes targeted by dysregulated miRNAs in hSOD1G93A muscle at each age from the miRSystem database

Available for download at
https://journals.biologists.com/dmm/article-lookup/doi/10.1242/dmm.052963#supplementary-data

Table S17. Enriched pathways of genes targeted by dysregulated miRNAs in hSOD1G93A muscle at 90 days old from the miRWalk database

Available for download at
https://journals.biologists.com/dmm/article-lookup/doi/10.1242/dmm.052963#supplementary-data

Table S18. Enriched pathways of genes targeted by dysregulated miRNAs in hSOD1G93A muscle at 120 days old from the miRWalk database

Available for download at
https://journals.biologists.com/dmm/article-lookup/doi/10.1242/dmm.052963#supplementary-data

Table S19. Enriched pathways of genes targeted by dysregulated miRNAs in hSOD1G93A muscle at 150 days old from the miRWalk database

Available for download at
https://journals.biologists.com/dmm/article-lookup/doi/10.1242/dmm.052963#supplementary-data

Disease Models & Mechanisms • Supplementary information

Table S20. List of PCR primers used in this study

Gene	Forward primer (5' to 3')	Reverse primer (5' to 3')
Tbp	CACGGACAACTGCGTTGATT	TGGATTGTTCTTCACTCTTGGC
Pgm1	CATCCCGACCCCAATCTCAC	TCATGTTTCGATCCCCGTCAC
Tpi1	TCACCGAGAAGGTCGTGTTC	CCAGTCACAGAACCTCCATA
Pgk1	TTGTGCATTGTAGAGGGCGT	TGACGAAGCTAACCAGAGGC
Eno3	ATTGCCTGCTCCTGAAGGTC	CACCAGTCTTGATCTGTCC
Pkm	CACCCTGGACAACGCTTACA	ATCTCATCAAACCTGCGGAC
Ldha	CAGGCTCCCCAGAACAAGATT	TCATCCGCCAAGTCCTTCATT
Aldh2	TTCCACAGGGGAGGTCATCT	CAGCCAGCGTAATAGCGGAG
Fbp2	ATTTCGGGGAGCGTGAATGT	GCTCATCCTCCGTGGTCTTT
Pik3r1	TATGCCTGCTCCGTAGTGGTA	TTGGGTGGCTTGAAGGTGAG
Prkaa2	GAGGTCATCTCAGGAAGGCTGT	CAAGCTGGTCTTGAGGGTCA
Hdac4	CTCTACATGTCCCTGCACCG	CAGAGGCTCAAGCTCGTTTC
